# Blood and CSF chemokines in Alzheimer’s disease and mild cognitive impairment: a systematic review and meta-analysis

**DOI:** 10.1186/s13195-023-01254-1

**Published:** 2023-06-08

**Authors:** Futao Zhou, Yangyan Sun, Xinhua Xie, Yushi Zhao

**Affiliations:** 1 School of Basic Medicine, Gannan Medical University, Ganzhou City, Jiangxi Province 341000 China; 2 Key Laboratory of Prevention and Treatment of Cardiovascular and Cerebrovascular Diseases of Ministry of Education, Gannan Medical University, Ganzhou, Jiangxi 341000 China

**Keywords:** Chemokine, Alzheimer’s disease, Mild cognitive impairment, Meta-analysis

## Abstract

**Objective:**

Chemokines, which are chemotactic inflammatory mediators involved in controlling the migration and residence of all immune cells, are closely associated with brain inflammation, recognized as one of the potential processes/mechanisms associated with cognitive impairment. We aim to determine the chemokines which are significantly altered in Alzheimer’s disease (AD) and mild cognitive impairment (MCI), as well as the respective effect sizes, by performing a meta-analysis of chemokines in cerebrospinal fluid (CSF) and blood (plasma or serum).

**Methods:**

We searched three databases (Pubmed, EMBASE and Cochrane library) for studies regarding chemokines. The three pairwise comparisons were as follows: AD *vs* HC, MCI *vs* healthy controls (HC), and AD *vs* MCI. The fold-change was calculated using the ratio of mean (RoM) chemokine concentration for every study. Subgroup analyses were performed for exploring the source of heterogeneity.

**Results:**

Of 2338 records identified from the databases, 61 articles comprising a total of 3937 patients with AD, 1459 with MCI, and 4434 healthy controls were included. The following chemokines were strongly associated with AD compared with HC: blood CXCL10 (RoM, 1.92, *p* = 0.039), blood CXCL9 (RoM, 1.78, *p* < 0.001), blood CCL27 (RoM, 1.34, *p* < 0.001), blood CCL15 (RoM, 1.29, *p* = 0.003), as well as CSF CCL2 (RoM, 1.19, *p* < 0.001). In the comparison of AD with MCI, there was significance for blood CXCL9 (RoM, 2.29, *p* < 0.001), blood CX3CL1 (RoM, 0.77, *p* = 0.017), and blood CCL1 (RoM, 1.37, *p* < 0.001). Of the chemokines tested, blood CX3CL1 (RoM, 2.02, *p* < 0.001) and CSF CCL2 (RoM, 1.16, *p* = 0.004) were significant for the comparison of MCI with healthy controls.

**Conclusions:**

Chemokines CCL1, CCL2, CCL15, CCL27, CXCL9, CXCL10, and CX3CL1 might be most promising to serve as key molecular markers of cognitive impairment, although more cohort studies with larger populations are needed.

**Supplementary Information:**

The online version contains supplementary material available at 10.1186/s13195-023-01254-1.

## Introduction

Alzheimer’s disease (AD) is the most common type of dementia, and it is on the rise among the older people [1]. It is one of the severe neurodegenerative diseases, with symptoms of diminished quality of life or disability. The pathological hallmarks in the AD brain are amyloid-β (Aβ)-derived plaques and tau-derived tangles. Based on accumulating evidence that Aβ overproduction leads to AD, the amyloid cascade hypothesis is widely accepted, and Aβ accumulation is believed to be the primary initial event that ultimately results in neuronal damage. Despite numerous clinical trials of treatments for AD that aimed to clear Aβ from the brain, to date no amyloid-targeting therapy has been successful in preventing or slowing the progression of cognitive impairment in symptomatic AD [2]. This suggests that while amyloid accumulation may be a key initiator of starting the pathological process, other downstream events such as neuroinflammation [3] and tau accumulation may be the predominant drivers of neurodegeneration [4]. Particularly, along with the discovery of elevated levels of inflammatory markers in AD, neuroinflammation has emerged as a vital player in AD pathogenesis [5].

Besides neuronal dysfunction, inflammation and glial activation are also well-known features of AD pathogenesis. Before being diagnosed with dementia, patients undergo a phase called as mild cognitive impairment (MCI), an intermediate status between normal aging and dementia [6]. Around neuritic plaques, activated microglia and reactive astrocytes, as well as their density, increase in proportion to the degree of neuronal injury [7, 8]. Inflammatory responses play an important role in the neurodegenerative cascades according to mounting data, and some biomarkers related to inflammation have tracking and detection accuracy for disease severity and progression [9, 10]. Biofluid-based markers such as P-tau and neurofilament light chain have gained much attention for their potential diagnostic and prognostic ability [11, 12]. A growing body of evidence highlights that chemokines, as mediators of neuroinflammation, play a critical role in the pathogenesis of cognitive impairment [13].

Chemokines are a type of cytokine involved in chemotaxis. They are heparin-binding proteins with molecular weights ranging from 7 to 15 kD. Chemokines are categorized into four subcategories based on the number and position of conserved NH2-terminal cysteine residues: CXC, CC, CX3C, and XC [14]. A number of cells, including leukocytes and neurons, can release chemokines. Functionally, chemokines may be pro-inflammatory or homeostatic. Binding to receptors, chemokines exert a key role in ensuring brain function by stimulating crosstalk between neurons, glial cells, and peripheral immune cells in physiological processes [15]. During inflammation, chemokines are upregulated and their most described feature is the chemoattraction of immune cells from the periphery to the brain, which in turn maintains inflammation through chemokine secretion [16, 17]. Apart from the well-documented role in the immune system, the chemokine/receptor system may participate in important pathophysiological processes in the central nervous system [18]. Accumulating evidence suggest that AD is associated with altered levels of chemokine biomarkers [19–21], and chemokines are considered to have either beneficial or detrimental effects upon nervous function by activating resident microglia and astrocytes and by inducing the release of inflammatory factors [22].

Some studies found that increased levels of circulating chemokines were linked to Alzheimer’s pathogenesis and can be used as biomarkers to track disease progression [23–25]. Other investigations, on the other hand, have reported null relationships of chemokine levels with AD [26, 27] or MCI [28, 29]. Chemokine marker differentiation performance is relatively poorly studied [20, 30], varies widely [31–33], and lacks a thorough analysis [34]. Therefore, we conducted a systematic review and meta-analysis using a widely applicable method of generating fold-changes in mean chemokine concentrations (i.e., ratio of mean) to identify available data on CSF and serum/plasma levels of all chemokines reported in patients with AD and MCI, and to determine which ones have significant and larger effect sizes among the predetermined groups.

## Methods

### Search strategy

With a registration number of CRD42022293988, the protocol for this systematic review has been prospectively recorded in the PROSPERO database. This systematic review and meta-analysis was performed according to the PRISMA guidelines [35]. We searched the databases (PubMed, EMBASE, and Cochrane Library) for relevant studies published from inception to December 15, 2021, to identify data on chemokines in CSF and plasma (or serum) among patients with AD or MCI and cognitive healthy controls (HC). Many different nomenclatures of chemokines were used for the search method due to the uneven naming format of chemokines in public publications. We used the following terms: chemokine*, ccl, cxcl, cx3cl, ccr, cxcr; dementia, Alzheimer*, cognit*, and so on, and screened titles and abstracts in the three databases. Meanwhile, relevant studies meeting the inclusion criteria were found in the reference lists of all included publications and review articles on the issue. The entire search strategy has been described in Table S1, in Supplemental file.

### Study selection

Relevant peer-reviewed articles reporting chemokine concentrations in living humans, published in either English or Chinese, were included if they matched the following criteria: (1) Data from at least two of the cohorts (AD, MCI, and control) were presented in original studies; (2) sample sources and essential data (N, mean, and standard deviation) were available; (3) the methods employed to diagnose AD and MCI in these studies were well-established; and (4) cognitively healthy subjects as controls. CSF chemokine concentrations were studied separately.

Articles were excluded if they involved neither AD nor MCI cohorts; had chemokine data from blood cells, brain tissues (or microvessels); used non-quantitative methods to assess chemokine concentrations (e.g., explorative proteomics or western blot); had a cohort with a mix of AD and MCI; without properly referenced methods, which we accepted as a well-established routine analysis; contained previously published data; studies measuring chemokine mRNA levels; in the control cohorts participants having an inflammatory, neurological, or psychiatric illness or symptom that would alter CSF or blood chemokine concentrations. In longitudinal cohorts, we considered the baseline data with the longest follow-up period. Meeting abstracts, case reports, review papers, and non-English and non-Chinese articles were excluded, as were studies with insufficient data, no clinical data (animal).

### Data extraction and statistical analysis

Two authors (ZF and SY) independently screened and retrieved papers based on the eligibility criteria, and four authors (ZF, SY, ZY, and XX) carefully reviewed and selected articles. Name of first author, year of publication, sample size, mean age or range, female sex ratio (percent), chemokine assay method, sample source (serum, plasma, or CSF), and AD/MCI diagnosis criteria were extracted for each study. Values of *n* and standard error (SE) or standard deviation (SD) were also extracted from each article. If the SE rather than SD was presented, it was converted to SD. We used a random effects model to pool the effect sizes from studies that reported two AD cohorts (such as mild-moderate AD and severe AD), and the pooled result was used as the study’s estimate. When median and interquartile range (IQR) or range were used as measures, we calculated the mean using a method provided by Wan et al. [36] and the SD using another method described by Luo et al. [37] based on sample size and median, IQR, or minimum/maximum values. To improve the normality of result distributions, a log transformation was employed.

In different laboratories, the cutpoints of chemokine levels were set based on a variety of ways. As a result, to reduce the variability in chemokine concentrations between laboratories and tests, a measure of fold-change between comparison (ratio of mean chemokine concentration, i.e., RoM) was used. Each RoM was generated in the context of a separate investigation, the corresponding 95% confidence intervals (CIs) was calculated using the delta method [38]. We used ratios of AD to controls, of MCI to controls, and of AD to MCI to do stepwise meta-analyses. A ratio above one implies that the chemokine concentration is higher in the former than the latter in the comparison, whereas a ratio less than one predicts the opposite. In this study, RoM values of 1.08 to 1.19, 1.20 to 1.32, and more than 1.32, or of 0.93 to 0.84, 0.83 to 0.76, and less than 0.76 (values derived from the corresponding reciprocals), are considered small, moderate, and large effect sizes, respectively [39].

A sensitivity analysis was performed to evaluate the impact of each study on the pooled effect size by removing one study at a time. The Newcastle–Ottawa Scale (NOS) was used to assess study quality. The *Q* test and the *I*^2^ statistic were used to test heterogeneity across studies. To obtain more conservative estimates, random effects meta-analyses were performed using the method of DerSimonian and Laird, with the estimate of heterogeneity derived using the inverse variance model. Publication bias was assessed by the Egger’s and Begg’s tests, as well as by viewing the symmetry of the funnel plot. When publication bias existed, the Trim-and-filled method was used to test and adjust for possible publication bias. Significance was defined as a *p* value of less than 0.05, and Bonferroni method was used for multiple comparison correction. Stata version 12.0 software (Stata Corp, College Station, Texas) was used in all the analyses.

## Results

**Fig. 1:**
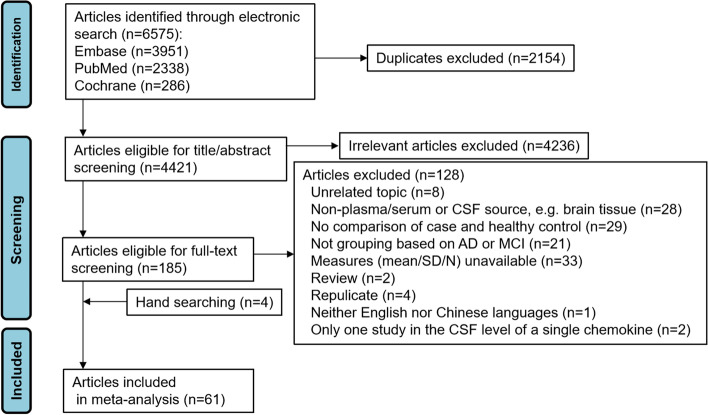
PRISMA diagram of study flow

The original search generated 4421 hits after duplicates were removed (2338 from PubMed, 3951 from Embase, and 286 from Cochrane). Titles and abstracts of the retrieved records were screened carefully using eligibility criteria to determinate their appropriateness. Thus, a total of 4236 irrelevant articles were excluded. After reviewing the full text of remaining articles (*n* = 185), 57 were deemed eligible for inclusion. Four publications [40–43] were hand-searched according to reference lists of related articles. In total, 61 articles were included in this meta-analysis. These studies yielded data from 3937 patients with AD, 1459 individuals with MCI, and 4434 healthy control subjects. There were 59 articles published in English and 2 in Chinese. Of 61 articles, 41 used case–control study designs, 12 were cross-sectional, and 8 were prospective cohort studies (Fig. 1, Table 1, Tables S1-2 in supplementary file). In most studies, the National Institute of Neurological and Communicative Disorders and Stroke/Disease Alzheimer’s and Related Disorders Association or DSM-IV criteria were used for AD diagnosis; for MCI diagnosis, the Petersen method was used in most studies.

**Table 1:** Summary characteristics of studies included in systematic review and meta-analysis

Ref	Study	Case/ctrl	*N*	Age	% F	Chemokines tested	Assay method	Study design	Sample	NOS	AD/MCI diagnosis
[44]	Alsadany, 2013	AD	25	72.2 (5.9)	56	IL-8	ELISA	Case–control	Plasma	7	NINCDS-ADRDA
		HC	25	72.8 (4.1)	52						
[45]	Andrés-Benito, 2020	AD	19	64 (8.6)	82	CXCL12 (SDF-1)	ELISA	Case–control	CSF	7	NR
		HC	70	67 (10.6)	81						
[46]	Blasko, 2006	AD	23	71.9 (9.6)	65	MCP-1	ELISA	Case–control	CSF	7	NINCDS-ADRDA
		HC	27	66.9 (9.4)	52						
[47]	Boccardi, 2019	MCI	73	77.5 (6.3)	61.6	MCP-1	ELISA	Case–control	Serum	7	Petersen
		HC	87	75.9 (9)	59.8						
[48]	Bonaccorso, 1998	AD	15	55.2 (22.2)	47	IL-8	ELISA	Case–control	Plasma	7	DSM-III-R
		HC	31	75.6 (9.1)	55						
[49]	Bonotis, 2008	AD	49	75.8 (6.2)	57.1	IL-8	ELISA	Case–control	Serum	7	NINCDS-ADRDA
		HC	21	71.2 (4.4)	52.4						
[40]	Björkqvist, 2012	AD	142	76 (56–87)	28	CCL18, CCL5, CCL7, CXCL8, CCL15	Quantibody Array	Case–control	Plasma	7	NINCDS-ADRDA
		HC	174	74 (62–99)	67						
[32]	Choi, 2008	AD	11	73.5 (4.0)	81.8	IL-8, IP-10, MCP-1 (+ CSF), RANTES, Eotaxin, MIP-1α	Luminex xMAP™	Case–control	CSF/serum	7	NINCDS-ADRDA
		HC	13	68.5 (7.2)	61.5						
[50]	Corrêa, 2011	AD	22	74.7 (10.2)	77.3	MCP-1, CXCL8, CXCL10	ELISA	Case–control	CSF	7	NINCDS-ADRDA
		HC	27	64.4 (11.1)	44.5						
[51]	Corsi, 2011	AD	70	75.6 (7.2)	61	IL-8, MCP-1	Biochip Array	Case–control	Plasma	7	NINCDS-ADRDA
		HC	6	73.4 (1.1)	67						
[52]	Delaby, 2015	AD	24	70.8 (8.7)	52	IL-8, MCP-1, MIP-1β, MIP-3β, RANTES, GRO-α, GRO-γ	ECL/ELISA	Case–control	CSF/ serum	7	NINCDS-ADRDA
		HC	31	66.6 (13.3)	67						
[53]	Dong, 2018	AD	26	66.6 (52–83)	69.2	IL-8, MCP-1	Luminex xMAP™	Prospective cohort	Serum	8	NINCDS-ADRDA
		MCI	16	71.0 (53–84)	50						
		HC	22	71.6 (60–88)	72.7						
[54]	Faura, 2020	AD	36	77 (72.5–83)	66.7	CCL23	ELISA	Cross-section	Serum	8	NINCDS-ADRDA
		HC	11	77 (72.5–83)	66.7						
[55]	Fenoglio, 2004	AD	269	75 (51–101)	71	MCP-1	ELISA	Case–control	Serum	7	NINCDS-ADRDA
		HC	203	72 (46–96)	54.2						
[56]	Galimberti, 2006	AD	94	79.1 (7)	75	MCP-1	ELISA	Case–control	Serum	7	NINCDS-ADRDA
		MCI	48	74.7 (6.8)	60						
		HC	24	71.5 (9.8)	29						
[57]	Galimberti, 2006	AD	22	59.4 (4.2)	59	IP-10, IL-8, MCP-1	ELISA	Case–control	CSF	7	NINCDS-ADRDA
		MCI	38	68 (10.5)	63						
		HC	41	64 (29.5)	63						
[58]	Gongora-River, 2019	AD	29	75.3 (7.3)	82.8	CCL27, CXCL12, CCL7, CXCL9	Luminex xMAP™	Case–control	Serum	8	NINCDS-ADRDA
		HC	49	72.85 (6.6)	75.5						
[59]	Grewal, 2016	MCI	45	76.2 (8.1)	100	MCP-1, eotaxin	Luminex xMAP™	Case–control	Plasma	7	DSM-V
		HC	30	72.97 (8.8)	100						
[60]	Gupta, 2017	AD	92	77.0 (7.4)	57	CCL1	ECL	Cross-section	Plasma	7	NINCDS-ADRDA
		MCI	65	74.8 (7.5)	55						
		HC	554	69.79 (6.5)	60						
[61]	Hazen, 2020	AD	154	74.9 (7.4)	58.4	CCL2, CCL4	Luminex xMAP™	Prospective cohort	Serum	7	NINCDS-ADRDA
		MCI	88	71.3 (10.3)	47.7						
[62]	He, 2017	AD	19	71.1 (35.4)	47.4	CCL1, CCL2, CCL3, CCL4, CCL7, CCL8, CCL11, CCL13, CCL15, CCL17, CCL19, CCL20, CCL21, CCL22, CCL24, CCL26, CCL27, CX3CL1, CXCL1, CXCL5, CXCL9, CXCL10, CXCL11, CXCL12, CXCL13, CXCL8	Luminex xMAP™	Case–control	Plasma	6	NINCDS-ADRDA
		HC	19	70.2 (24.1)	47.4						
[63]	Hesse, 2016	AD	41/36	68(9.5)/68 (5.9)	71/69	IL-8	ECL	Case–control	CSF /serum	7	NINCDS-ADRDA
		HC	23/24	69(11.6)/70(9.5)	57/50						
[28]	Hochstrasser, 2011	AD	92	78.8 (7.1)	77.2	CCL4, CCL2, CCL22, CCL15, CXCL9	ELISA	Case–control	Plasma	7	NINCDS-ADRDA /Petersen
		MCI	67	73.8 (8)	65.7						
		HC	40	72.2 (6.3)	52.5						
[25]	Kim, 2008	AD	51	78.2 (6.1)	82.4	CX3CL1	ELISA	Cross-section	Plasma	8	NINCDS-ADRDA
		MCI	51	74.6 (7.0)	80.4						
		HC	57	70.5 (3.8)	70.2						
[64]	Kim, 2011	AD	18	75.9 (6)	50	MCP-1, IL-8	ELISA	Cross-section	Plasma	7	NINCDS-ADRDA
		MCI	20	76.1 (2.8)	45						
		HC	21	75.5 (1.3)	52.4						
[65]	King, 2019	MCI	38	75.6 (1.2)	34	IL-8	ECL	Cross-section	Plasma	7	NIA-AA
		HC	20	75.9 (1.6)	20						
[66]	Kulczyńska -Przybik, 2020	AD	42	72.5 (51–89)	NR	CCL2, CX3CL1	ELISA	Cross-section	CSF /serum	7	NIA-AA
		MCI	18	72.5 (51–89)	NR						
		HC	20	72.5 (51–89)	NR						
[67]	Laske, 2008	AD	30	70.5 (8.2)	60	CXCL12	ELISA	Case–control	Plasma	7	NINCDS-ADRDA
		HC	30	69.9 (11.1)	33.3						
[43]	Lee, 2008	AD	10	82.7 (8.4)	70	CCL27, CXCL1, CCL7, CXCL12, CXCL9	BPC	Case–control	Plasma	7	NINCDS-ADRDA
		MCI	25	72.1 (5.6)	64						
		HC	19	71.4 (5.3)	68.4						
[23]	Lee, 2018	AD	310	80.1 (7.2)	44.8	MCP-1	Luminex xMAP™	Prospective cohort	Plasma	7	NIA-AA
		MCI	66	75.4 (8.2)	47						
		HC	120	74.9 (7.8)	45.8						
[41]	Leung, 2013	AD	117	76.2 (6.1)	66.7	IL-8, Eotaxin-1, IP-10, MCP-1	Bio-Plex Luminex 200	Prospective cohort	Plasma	7	NR
		MCI	122	739 (5.6)	49.2						
		HC	112	72.3 (6.7)	53.6						
[68]	Li, 2008	AD	138	78.3 (5.9)	39	MIP-1α	ELISA	Case–control	Serum	8	DSM-III-R
		HC	180	69.7 (4.2)	31						
[31]	Liang, 2021	AD	28	78.3 (8.8)	75	IL-8, IP-10, MCP-1, RANTES, CCL3, CCL4, Eotaxin-1	ECL	Cross-section	Plasma	7	NIA-AA
		MCI	51	75.6 (8.6)	78.4						
		HC	12	66.3 (5.9)	75						
[69]	Llano, 2012	AD	15	22.1 (35.9)	20	IL-8	ECL	Cross-section	CSF	7	NINCDS-ADRDA
		HC	7	29.1 (3.5)	29						
[70]	Lourenco, 2021	AD	14	67.8 (4.8)	71	IL-8, IP-10, MCP-1, RANTES, MIP-1α	ELISA	Cross-section	CSF	7	NR
		MCI	14	71.6 (5.9)	43						
		HC	25	67.8 (4.8)	60						
[71]	Magaki, 2007	AD	7	79.14 (0.99)	57	IL-8	ELISA	Case–control	Serum	7	DSM-IV/Petersen
		MCI	31	75.1 (1.3)	52						
		HC	21	73.1 (1.6)	67						
[72]	Magdalinou, 2015	AD	26	62.8 (7.7)	65.4	MCP-1	Immunoassays	Prospective cohort	CSF	7	NINCDS-ADRDA
		HC	30	59.8 (9.8)	50						
[29]	Marksteiner, 2011	AD	96	77.0 (0.8)	NR	IL-8, MCP-3, MIP1δ, MIP4, RANTES	ELISA	Case–control	Plasma	7	NINCDS-ADRDA/Petersen
		MCI	44	73.5 (1.2)	NR						
		HC	19	72.1 (1.3)	42						
[73]	Mattsson, 2011	AD	25	74 (4)	56	IL-8, MCP-1	ELISA	Cross-section	CSF	7	NINCDS-ADRDA
		MCI	13	71 (4)	62						
		HC	19	74 (5)	53						
[33]	Mohd, 2017	AD	39	80.7 (6.4)	56	IL-8, CXCX1, CXCL10, MCP-1, MIP-1α	ELISA	Case–control	Serum	7	NINCDS-ADRDA
		HC	39	72.1 (5.04)	38						
[20]	Morgan, 2019	AD	262	75.9 (6.2)	NR	MCP-1, Eotaxin-1, MIP-1β	ECL	Case–control	Plasma	7	NINCDS-ADRDA
		MCI	199	74.8 (5.8)	NR						
		HC	259	72.9 (6.7)	NR						
[74]	Nordengen, 2019	AD	27	67.6 (5.2)	48	MCP-1, fractalkine	QuickPlex SQ 120 system	Case–control	CSF	7	NIA-AA
		MCI	40	66.6 (7.4)	57						
		HC	36	61.1 (9.2)	53						
[26]	O’Bryant, 2016	AD	79	76.1 (8.6)	38	CCL26, CCL17, CCL1	ECL	Case–control	Plasma	7	NINCDS-ADRDA
		HC	65	71.2 (9.2)	53						
[30]	Pedrini, 2017	AD	67	76 (7)	66	MCP-1, EOTAXN3, TARC	ECL	Prospective cohort	Plasma	7	NINCDS-ADRDA
		MCI	39	75 (6)	44						
		HC	559	69 (6)	58						
[21]	Perea, 2018	AD	14	68 (4.2)	64.3	Fractalkine	ELISA	Case–control	CSF /serum	7	NINCDS-ADRDA
		MCI	14	70 (3.52)	64.3						
		HC	14	64 (2.9)	50						
[75]	Porcellini, 2013	AD	291	75.1 (8)	NR	MCP-1	BPC	Case–control	Plasma	7	NINCDS/ADRDA
		HC	148	71.6 (4.7)	NR						
[76]	Rauchmann, 2020	AD	188	74.7 (7.5)	43	IP-10	ECL	Case–control	CSF	7	NINCDS-ADRDA/NIA-AA
		HC	94	75.1 (7.1)	38.3						
[77]	Reale, 2012	AD	38	73.8 (5.5)	47.4	MCP-1, RANTES	ELISA	Case–control	Plasma	7	NINCDS-ADRDA
		HC	39	72.7 (4.8)	53.8						
[78]	Rosén, 2014	AD	25	67.2 (17.5)	64	MCP-1	ELISA	Case–control	CSF	7	NINCDS-ADRDA
		HC	25	60.1 (8.7)	64						
[79]	Schipke, 2019	AD	81	81.9 (7.8)	67	MCP-1	ELISA	Case–control	Serum	7	NINCDS-ADRDA
		HC	79	64.5 (2.7)	35						
[80]	Shi, 2011	AD	50	68.1 (9.5)	38	Fractalkine	ELISA	Cross-section	CSF	7	NINCDS-ADRDA
		HC	137	58.9 (18.4)	45						
[42]	Soares, 2009	AD	19	81.0 (4.8)	63.2	RANTES, IL-8	Luminex xMAP	Prospective cohort	Plasma	7	NINCDS-ADRDA
		HC	22	76.5 (7.5)	63.6						
[27]	Villarrea,2015	AD	28	81.9 (9.2)	78.6	I309, CCL17, CCL26, CCL3	ECL	Cross-section	Serum	7	NINCDS-ADRDA
		MCI	30	81.2 (7.8)	66.7						
		HC	77	76.5 (6.7)	64.9						
[81]	Wennström, 2015	AD	49	77.1 (6)	76	MCP-1, IL-8, IP-10	ELISA	Case–control	CSF	7	DSM-III-R
		HC	44	63.7 (10.3)	52						
[24]	Westin, 2012	AD	47	74 (6)	21	CCL2, CCL11, CCL13, CCL26	ELISA	Prospective cohort	CSF /plasma	7	NINCDS-ADRDA
		MCI	52	64 (9)	46						
		HC	30	72 (8)	57						
[82]	Wu, 2015	AD	41	73.1 (9.4)	65.9	Fractalkine	ELISA	Case–control	Plasma	7	NINCDS
		HC	40	63.0 (5.6)	67.5						
[83]	Xu, 2021	AD	212	73.4 (8.5)	55.7	MCP-1	Luminex xMAP™	Case–control	Serum	7	NINCDS-ADRDA
		HC	268	73.2 (8.7)	55.2						
[84]	Yu, 2005	AD	11	65.5 (11.9)	36	IL8	ELISA	Case–control	CSF /serum	6	NINCDS-ADRDA
		HC	13	63.7 (12.8)	46						
[85]	Zhang, 2008	AD	48	70 (9)	40	IL-8	Immunobead-based multiplex	Case–control	CSF	7	NINCDS-ADRDA
		MCI	12	71 (12)	33						
		HC	95	63 (12)	54						
[86]	Zhang, 2013	AD	24	77.9 (7.7)	58.5	MCP-1	ELISA	Case–control	Plasma	7	NINCDS-ADRDA
		HC	31	75.4 (9.5)	48.4						
[87]	Zhu, 2017	AD	96	77.3 (7.3)	62.5	IL-8	Luminex xMAP™	Case–control	Serum	7	NINCDS-ADRDA
		MCI	140	71.23 (8.1)	47.9						
		HC	79	68.3 (6.0)	51.9						

*Ref* Reference, *AD* Alzheimer’s disease, *MCI* Mild cognitive impairment, *ELISA* Enzyme-linked immunosorbent assay, *ECL* Electrochemiluminescence, *BPC* Bio-Plex cytokine assay, *CCL1* I-309, *CCL2* MCP-1, *CCL3* MIP-1α, *CCL4* MIP-1β, *CCL7* MCP-3, *CCL8* MCP-2, *CCL11* Eotaxin-1, *CCL13* MCP-4, *CCL15* MIP-1δ, *CCL17* TARC, *CCL19* MIP-3β, *CCL20* MIP-3α, *CCL22* MDC, *CCL24* Eotaxin-2, *CCL26* Eotaxin-3, *CCL27* CTACK, *CX3CL1* Fractalkine, *CXCL1* GRO, *CXCL5* ENA-78, *CXCL9* MIG, *CXCL10* IP-10, *CXCL11* I-TAC, *CXCL12* SDF-1, *CXCL8* IL-8, *NINCDS-ADRDA* National Institute of Neurological and Communicative Diseases and Stroke/Alzheimer’s Disease and Related Disorders Association, *DSM-III-R* Diagnostic and Statistical Manual of Mental Disorders-III-Revised, *NIA-AA* National Institute on Aging and Alzheimer’s Association, *CSF* Cerebrospinal fluid, *NR* Not reported

In terms of sample sources, 14 studies reported chemokines only from CSF, 15 only from serum, 25 only from plasma, and 7 from both blood and CSF. In addition, 35 studies used ELISA, 10 used Luminex, 8 used Electrochemiluminescence, and 8 used other methods to determine chemokine levels. There were 14 CC chemokines (CCL1, i.e., CC chemokine ligand 1; CCL2, CCL3, CCL4, CCL5, CCL7, CCL11, CCL15, CCL17, CCL18, CCL19, CCL22, CCL26, CCL27), 5 CXC chemokines (CXCL1, CXCL8, CXCL9, CXCL10, CXCL12), and only one CX3C chemokine (CX3CL1, i.e., fractalkine), with no report regarding the XC chemokines (Table 1). The studies included were considered as high quality (with NOS scores ranging from 6 to 8).

### Comparison between AD and HC in serum/plasma chemokine levels

We first meta-analyzed data on serum/plasma chemokine concentrations in AD versus HC. The following chemokines were investigated by two or more studies per chemokine: 14 CC motifs (see Table 2), 5 CXC motifs (CXCL1, CXCL8, CXCL9, CXCL10, CXCL12), the CX3C motif (fractalkine). Of these chemokines tested, only CCL2 (MCP-1) and CXCL8 (IL-8) had much more data for meta-analysis. These studies included 43 cohorts with AD and healthy controls, totaling 3225 patients and 3620 controls.

**Table 2:** Meta-analysis of studies regarding plasma/serum and CSF chemokines

Comparison	Chemokine	Sample	No of study	*N*	Main effect		Heterogeneity		Publication bias	
					RoM (95% CI)	*P* value	*I*^2^ (%)	*P* value	*p* for Begg’s	*p* for Egger’s
AD vs HC	CCL1	Plasma/ serum	3	199/696	1.56 (1.02–2.39)	0.126^#^	92.3	< 0.001	1	0.51
	CCL2		20	2017/1953	1.13 (0.92–1.39)	0.726^#^	98.7	< 0.001	0.02	0.06
	CCL3		5	244/321	1.36 (0.80–2.30)	0.759^#^	99.1	< 0.001	1	0.72
	CCL4		4	406/342	0.96 (0.82–1.12)	1.701^#^	37.9	0.148	0.31	0.09
	CCL5		6	216/136	0.99 (0.62–1.60)	2.73^#^	97.8	< 0.001	1	0.57
	CCL7		4	277/261	1.19 (0.83–1.72)	2.304^#^	87	0.349	1	0.76
	CCL11		3	301/284	1.17 (0.83–1.64)	1.131^#^	67.3	0.047	1	0.80
	CCL15		3	207/78	1.29 (1.13–1.47)	**0.009**^#^	48.7	0.142	0.09	0.06
	CCL17		4	140/175	1.31 (0.88–1.96)	0.18	80	0.002	0.31	0.60
	CCL18		2	238/193	1.05 (0.79–1.38)	0.753	62.9	0.101	-	-
	CCL19		2	43/50	1.71 (0.30–9.87)	0.55	91.2	0.001	-	-
	CCL22		2	111/59	1.04 (0.65–1.67)	0.864	72.7	0.056	-	-
	CCL26		3	174/701	0.81 (0.60–1.10)	0.346^#^	0	0.747	1	0.25
	CCL27		3	58/87	1.34 (1.19–1.51)	** < 0.001**	0	0.368	0.30	0.55
	CXCL1		4	92/108	1.28 (0.93–1.77)	0.126	55.1	0.083	0.31	0.41
	CXCL8		18	727/580	1.18 (0.85–1.62)	0.966^#^	98	< 0.001	0.77^a^	0.21^a^
	CXCL9		3	140/108	1.78 (1.39–2.28)	** < 0.001**	46.9	0.152	0.31	0.05
	CXCL10		3	78/64	1.92 (1.03–3.58)	**0.039**	99.4	< 0.001	1	0.44
	CXCL12		3	78/98	1.03 (0.85–1.24)	0.727	91	< 0.001	1	0.97
	CX3CL1		4	148/131	1.20 (0.96–1.50)	0.33^#^	84.1	< 0.001	1	0.51
AD vs MCI	CCL1		2	120/95	1.37 (1.17–1.59)	** < 0.001**	0	0.772	-	-
	CCL2		9	1026/573	1.13 (0.80–1.59)	1.095^#^	85.6	< 0.001	0.60	0.53
	CCL3		2	56/81	1.00 (0.86–1.16)	2.982^#^	0	0.722	-	-
	CCL4		4	536/405	1.04 (0.95–1.14)	1.227^#^	0	0.704	0.31	0.11
	CCL5		2	124/95	0.98 (0.77–1.25)	2.7^#^	52.5	0.147	-	-
	CCL7		2	106/69	1.21 (0.96–1.53)	0.3^#^	0	0.843	-	-
	CCL11		2	290/250	1.09 (0.96–1.24)	0.489^#^	32.1	0.225	-	-
	CCL15		2	188/111	1.09 (1.00–1.19)	0.135^#^	0	0.996	-	-
	CXCL8		6	271/302	1.13 (0.80–1.59)	1.497^#^	89.3	< 0.001	1	0.08
	CXCL9		2	46/61	2.29 (1.57–3.32)	** < 0.001**	25.8	0.246	-	-
	CX3CL1		2	93/69	0.77 (0.62–0.96)	**0.05**^#^	0	0.938	-	-
MCI vs HC	CCL1		2	95/631	1.30 (0.66–2.54)	1.356^#^	90.6	0.001	-	-
	CCL2		13	655/665	1.07 (0.98–1.16)	0.369^#^	77.6	< 0.001	0.50	0.412
	CCL3		2	95/31	0.64 (0.22–1.92)	1.827^#^	91	0.001	-	-
	CCL4		3	317/311	0.94 (0.83–1.07)	1.059^#^	1.8	0.361	1	0.51
	CCL5		2	81/89	1.28 (0.50–3.23)	1.281^#^	79.7	0.027	-	-
	CCL7		2	69/38	0.94 (0.73–1.22)	1.971^#^	42.1	0.189	-	-
	CCL11		6	347/331	1.03 (0.97–1.1)	0.933^#^	0	0.824	1	0.49
	CCL15		2	111/59	1.16 (1.01–1.35)	0.117^#^	0	0.654	-	-
	CCL26		2	82/107	1.27 (0.63–2.57)	1.768^#^	41	0.193	-	-
	CXCL8		7	340/194	1.05 (0.61–1.80)	2.589^#^	96.4	< 0.001	0.45^a^	0.79^a^
	CXCL9		2	61/52	1.01 (0.76–1.34)	2.79^#^	0	0.324	-	-
	CX3CL1		2	69/77	2.02 (1.58–2.58)	** < 0.001**	19.2	0.266	-	-
AD vs HC	CCL2	CSF	12	310/338	1.19 (1.13–1.25)	** < 0.001**	0	0.81	0.84	0.73
	CCL5		2	38/56	1.30 (0.66–2.57)	0.451	48.3	0.164	-	-
	CXCL8		10	260/308	1.22 (0.99–1.50)	0.174^#^	86.8	< 0.001	0.59	0.85
	CXCL10		5	295/231	1.05 (0.84–1.31)	2.007^#^	68.8	0.012	0.46	0.60
	CXCL12		2	42/47	0.95 (0.62–1.46)	0.803	83.8	0.013	-	-
	CX3CL1		4	133/207	1.09 (0.93–1.28)	0.933^#^	51.6	0.102	0.73	0.71
MCI vs HC	CCL2		6	175/171	1.16 (1.05–1.29)	**0.012**^#^	59.2	0.031	0.26	0.39
	CXCL8		3	65/85	1.52 (0.70–3.29)	0.585^#^	96.2	< 0.001	1	0.37
	CXCL10		2	52/66	1.19 (0.48–2.97)	2.103^#^	92.6	< 0.001	-	-
	CX3CL1		3	72/70	1.27 (0.71–2.28)	1.266^#^	89.7	< 0.001	1	0.63
AD vs MCI	CCL2		5	130/123	1.04 (0.90–1.19)	1.893^#^	56.3	0.058	1	0.33
	CXCL8		3	61/65	0.72 (0.38–1.37)	0.96^#^	94.7	< 0.001	1	0.47
	CXCL10		2	36/52	1.08 (0.56–2.08)	2.454^#^	76.2	0.04	-	-
	CX3CL1		3	83/72	0.80 (0.51–1.27)	1.044^#^	81.1	0.005	1	0.33

*AD* Alzheimer’s disease, *MCI* Mild cognitive impairment, *HC* Healthy control, *CSF* Cerebrospinal fluid, *RoM* Ratio of mean, *CI* Confident interval

^a^ When removing the outlier (Kim et al.’s study)

^#^*p* value with Bonferroni corrected

The serum/plasma ratios of AD to healthy controls were more than one in the following chemokines (Figure S13 in Supplementary file). In the CC motif, data on the chemokines (CCL1, CCL15, and CCL27) from two or three cohorts of AD and controls yielded average ratios of 1.56 (95% CI, 1.02–2.39, *p* = 0.042, corrected *p* = 0.126; *I*^2^ = 92.3%), of 1.29 (95% CI, 1.13–1.47, corrected *p* = 0.009; *I*^2^ = 48.7%), and of 1.34 (95% CI, 1.19–1.51, *p* < 0.001; *I*^2^ = 0%), respectively. In the CXC motif, the plasma/serum level of CXCL10 (IP-10) was significantly elevated in patients with AD compared with HC, with a large effect size (average ratio, 1.92; 95% CI, 1.03–3.58, *p* = 0.039; *I*^2^ = 99.4%) in 78 AD and 64 controls, and of CXCL9 did so (RoM, 1.78, 95% CI, 1.39–2.28, *p* < 0001; *I*^2^ = 46.9%) in 140 AD and 108 controls.

The levels of serum/plasma chemokine CXCL8 (IL-8) were reported by 18 studies, consisting 727 patients with AD and 580 healthy controls. The average AD to control ratio was 1.18 (95% CI, 0.85–1.62, corrected *p* = 0.966; *I*^2^ = 98%; Figure S13 in Supplementary file). After removing an outlier from Kim et al.’s study [64], the remaining data exhibited statistical significance with lower heterogeneity (RoM, 1.31, 95% CI, 1.11–1.55, corrected *p* = 0.009; *I*^2^ = 88%, *p* < 0.001).

Nineteen studies presented data on serum/plasma chemokine CCL2 (MCP-1), comprising 2017 patients with AD and 1953 healthy controls. CCL2 (MCP-1) concentrations in plasma/serum were not substantially different between AD and HC (average ratio, 1.13; 95% CI, 0.92–1.39, corrected *p* = 0.726; *I*^2^ = 98.7%, *p* < 0.001). The plasma/serum concentrations of the other chemokines tested (CCL3, CCL4, CCL5, CCL7, CCL11, CCL17, CCL18, CCL19, CCL22, CCL26; CXCL1, CXCL12; CX3CL1) had no significant differences between AD and HC (average ratios ranging from 0.81 to 1.71, *p* > 0.05).

### Comparison between AD and MCI in serum/plasma marker levels

In the comparison between AD and MCI, 20 articles reported serum or plasma levels of chemokines in 8 CC motifs (see Table 2), 2 CXC motifs (CXCL8 and CXCL9), and CX3CL1 (fractalkine). There were 20 AD versus 20 MCI cohorts, including 1651 AD patients and 1186 MCI subjects in the comparison.

In the CC motif, data from two cohorts of AD and MCI evaluating the chemokine CCL1 in plasma or serum, which included 120 AD patients and 95 MCI patients, revealed a substantial effect size (RoM, 1.37, 95% CI, 1.17–1.59, corrected *p* < 0.001; *I*^2^ = 0%; Table 2 and Figure S14 in Supplementary file). We found a large or huge effect size in CXCL9 (RoM, 2.29, 95% CI, 1.57–3.32, *p* < 0.001; *I*^2^ = 25.8%; Fig. 3), with a 130% rise in AD when compared to MCI. In addition, the chemokine CX3CL1 (fractalkine) concentrations in serum/plasma differed modestly and marginally (RoM, 0.77, 95% CI, 0.62–0.96, corrected *p* = 0.051; *I*^2^ = 0%; Fig. 3) between AD and MCI.

The concentrations of the other chemokines (CCL2, CCL3, CCL4, CCL5, CCL7, CCL11, CCL15, and CXCL8) in plasma or serum did not change substantially between AD and MCI (average ratios ranging from 0.98 to 1.13, all *p* > 0.05).

### Comparison between MCI and HC in serum/plasma chemokine levels

In the comparison between MCI and HC, 12 chemokines, including 9 CC motifs (see Table 2), 2 CXC motifs (CXCL8 and CXCL9), and 1 CX3C motif (fractalkine), were reported by 17 studies in 22 MCI and HC cohorts, totaling 1254 MCI patients and 2196 healthy controls.

The serum/plasma concentrations of the chemokine CX3CL1 (fractalkine) differed substantially between MCI and HC (RoM, 2.02, 95% CI, 1.58–2.58, *p* < 0.001; *I*^2^ = 19.2%). The chemokine CCL15 in plasma/serum from two MCI and HC cohorts, which included 111 patients with MCI and 59 HC, showed an average ratio of 1.16 (95% CI, 1.01–1.35, corrected *p* = 0.117; *I*^2^ = 0%; Fig. 3 and Figure S14 in Supplementary file).

Other chemokines in plasma/serum (CCL1, CCL2, CCL3, CCL4, CCL5, CCL7, CCL11, CCL26, CXCL8, and CXCL9) had no significant differences between MCI and HC (average ratios ranging from 0.64 to 1.28, all *p* > 0.05).

### Pairwise comparisons among AD, MCI, and HC in CSF chemokine levels

In CSF, the chemokines CCL2 (MCP-1), CCL5, CXCL8 (IL-8), CXCL10 (IP-10), CXCL12, and CX3CL1 (fractalkine) have available data in the literature from AD cohorts, MCI cohorts, or healthy control cohorts. Six chemokines had data in AD *vs* HC, 4 in AD versus MCI, and 4 in MCI versus HC. This included 21 AD cohorts and 21 control cohorts, as well as 8 MCI cohorts, totaling 743 AD patients, 201 MCI subjects, and 821 controls.

In CSF CCL2 (MCP-1), twelve studies on AD against control consisted of 310 AD patients and 338 controls, and six studies on MCI against control consisted of 175 MCI and 171 controls. In contrast to the lack of significance in serum/plasma, it was observed that the CSF levels of CCL2 were higher in AD than in controls (RoM, 1.19, 95% CI, 1.13–1.25, corrected *p* < 0.001; *I*^2^ = 0), and in MCI than HC (RoM, 1.16, 95% CI, 1.05–1.29, corrected *p* = 0.012; *I*^2^ = 59.2%). When comparing AD with MCI, however, the CSF CCL2 (MCP-1) concentrations did not differ substantially (RoM, 1.04, 95% CI, 0.90–1.19, corrected *p* = 1.893; *I*^2^ = 56.3%; Fig. 4), suggesting that CSF CCL2 appears to be a marker reflecting the degree of cognitive impairment, although it has a slight elevation.

With sufficient data from the most cohort studies among all the chemokines tested, there was no difference in CSF concentration of IL-8 (CXCL8) between AD patients and controls (average ratio 1.22, 95% CI, 0.99–1.50, corrected *p* = 0.174; *I*^2^ = 86.8%). The three pairwise comparisons for CSF CXCL10 (IP-10) and CX3CL1 (Fractalkine) had available data but did not yield statistically significant findings (*p* > 0.05, Table 2 and Figure S15 in Supplementary file). Other chemokines in CSF (CCL5, CXCL10, CXCL12, and CX3CL1) had no significant differences in the three pairwise comparisons (average ratios ranging from 0.80 to 1.52, all *p* > 0.05).

**Fig. 2:**
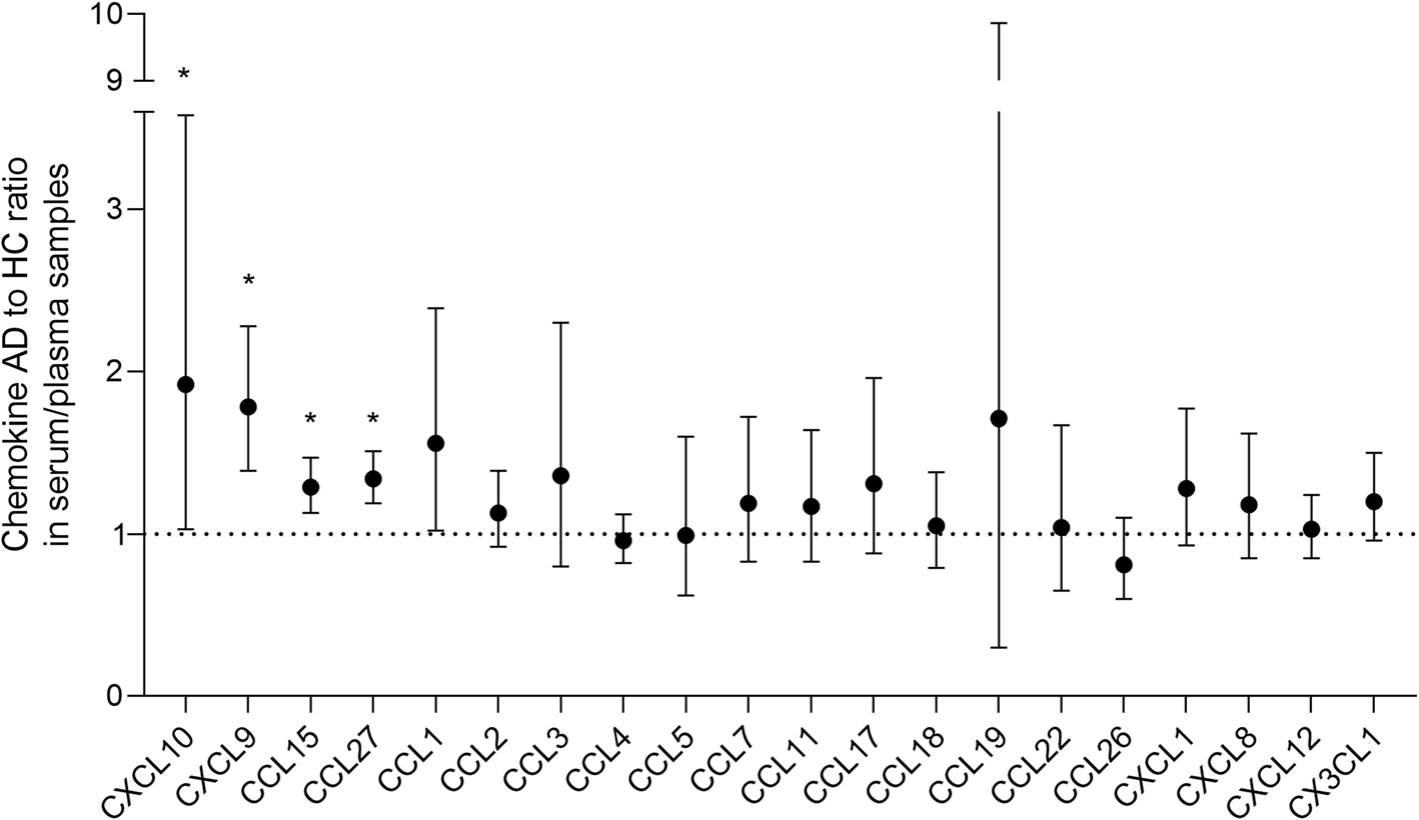
The performance of serum/plasma chemokines in differentiating Alzheimer’s disease from healthy controls. Based on average AD to control ratios, head-to-head chemokine performance in serum/plasma. An asterisk indicates significance, *p* < 0.05

**Fig. 3:**
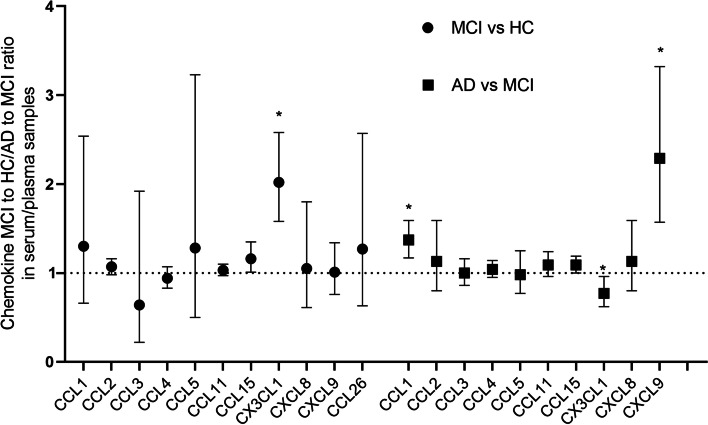
The performance of serum/plasma chemokines in differentiating MCI from healthy individuals and AD from MCI. The average ratios of MCI to controls and AD to MCI were used to compare chemokine performance in serum or plasma. An asterisk indicates significance, *p* < 0.05

**Fig. 4:**
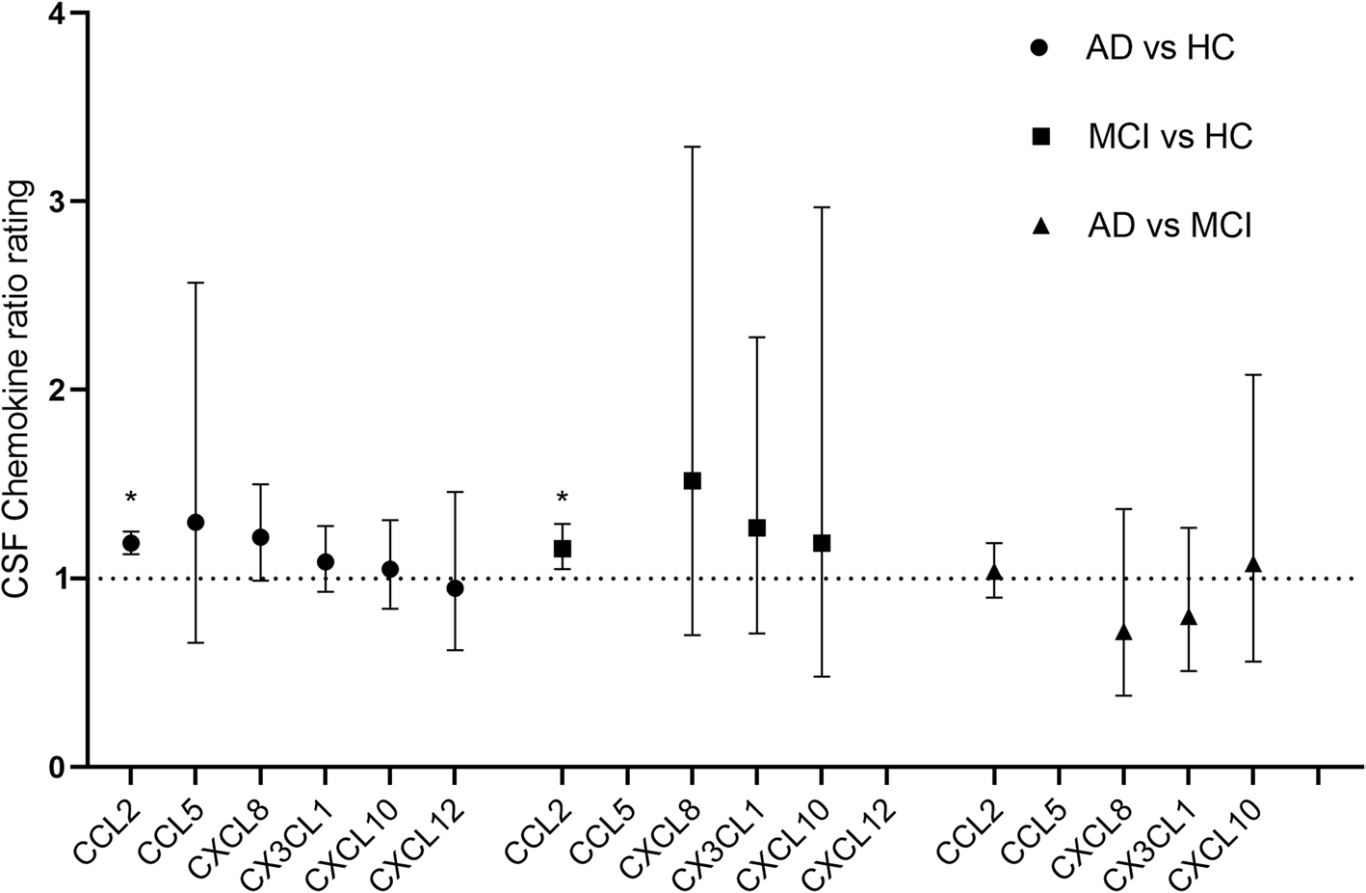
The ability of CSF chemokines to distinguish AD from MCI. Average AD to MCI ratios were used to compare CSF chemokine performance. An asterisk indicates significance, *p* < 0.05

**Fig. 5:**
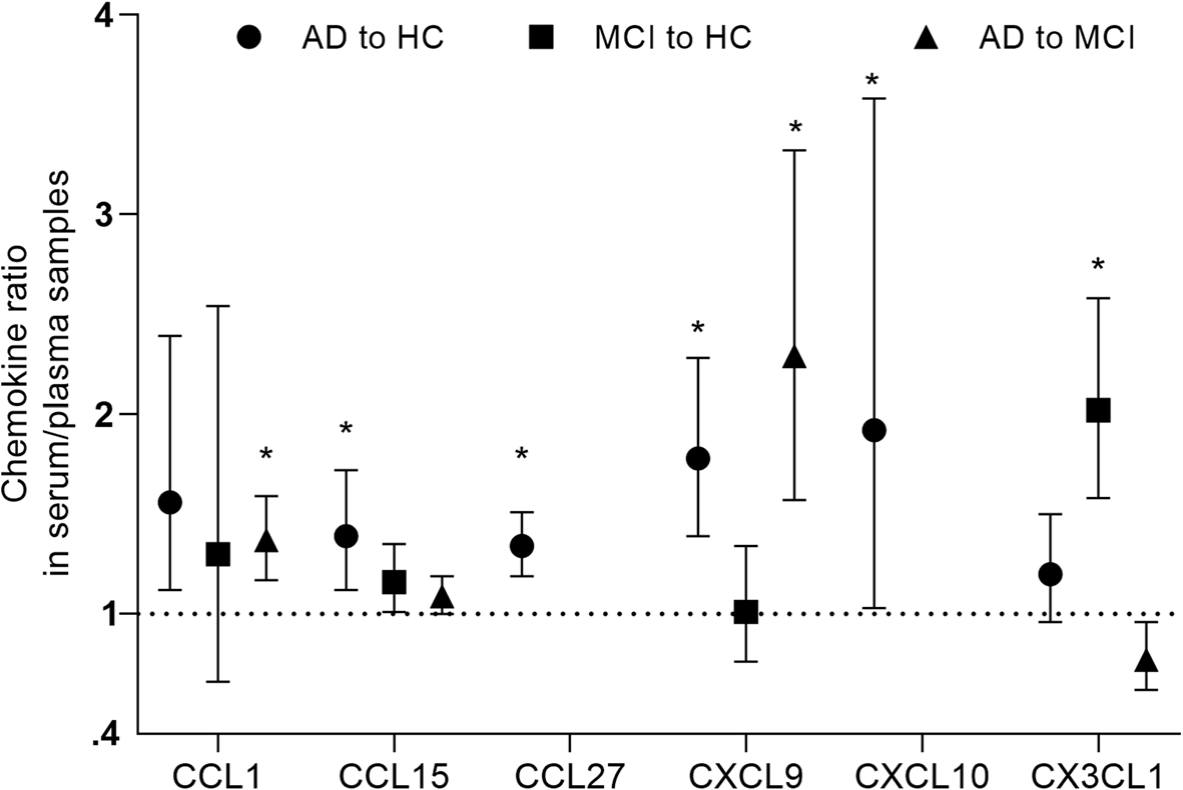
The performance of the chemokines CCL1, CCL15, CCL27, CXCL9, CXCL10, and CX3CL1 in the serum/plasma in the three pairwise comparisons. An asterisk indicates significance, *p* < 0.05

In summary, Figs. 2, 3, 4, and 5 exhibit head-to-head arrangement of chemokine performance. In the comparison between AD and HC, blood CXCL10, CXCL9, CCL27, and CCL15 were significant with good effect sizes. In the comparison between MCI and HC, blood CX3CL1 was significant with a large effect size. In the comparison between AD and MCI, blood CXCL9 and CCL1 had large effect sizes, and blood CX3CL1 was marginally significant with higher level in MCI compared with in AD. Among the chemokines investigated, only CSF MCP-1 was significant in both AD *vs* HC and MCI *vs* HC. None of the other CSF biomarkers were found to be significant in these comparisons.

### Subgroup analysis, study heterogeneity, and publication bias

There was considerable evidence of heterogeneity with *I*^2^ values up to 99.1% (see Figure S13-20 in Supplementary file). Subject to limited data availability, subgroup analyses were mainly conducted for CCL2 (MCP-1) and CXCL8 (Figures S16-20, Supplementary file). Sample source, study design, and assay method could account, partly, and to a lesser extent, for the source of heterogeneity.

According to sensitivity analysis, the performance of most chemokine biomarkers in peripheral blood and CSF was not substantially influenced by specific study. The funnel plots and Begg’s/Egger’s tests revealed that in the majority of the analyses, there was no significant publication bias (Figures S2-S12 in Supplementary file). However, publication bias was evident for the meta-analysis of blood CCL2 in AD *vs* HC (Begg's test *p* = 0.02, substantial asymmetry of the funnel plot). As a result, we used the Trim and Filled method to re-calculate the pooled estimate. The analysis suggested that the adjusted RoM (95% CI) was 1.13 (0.92–1.39), remaining without statistical significance (Figure S1 in supplementary file).

The sensitivity analysis demonstrated that in the analysis of blood CXCL8 in AD *vs* HC, an outlier from Kim et al.’s study [64] was the only one that would change significantly the pooled result (Figure S2 in Supplementary file). Moreover, the meta-analysis of blood CXCL8 in AD *vs* HC demonstrated publication bias (Egger’s test *p* = 0.04, significant funnel plot asymmetry, figure not shown). After the outlier was removed, however, publication bias was minimized (Begg’s test *p* = 0.77, Egger’s test *p* = 0.21). In addition, Kim et al.’s study on blood CXCL8 in MCI *vs* HC had also a significant influence on heterogeneity and publication bias, but failed to change the significance of the result.

## Discussion

### Principal findings and possible explanations

The current investigation examined the conflicting results of the studies on the serum/plasma and CSF chemokine markers linked to AD or MCI. The current findings show that AD is associated with higher blood levels of CCL15, CCL27, CXCL9, and CXCL10, and higher CSF levels of CCL2 compared with controls. Furthermore, blood levels of CXCL9 and CCL1 are higher in AD compared with MCI, and blood CX3CL1 (Fractalkine) has a higher level in MCI compared with controls. This includes reporting a wide range of changes in blood chemokines, such as a 90% higher CXCL10 level, an almost 80% higher CXCL9 level, and 29–35% higher CCL15/CCL27 levels in AD *vs* controls; an over twice higher CX3CL1 level in MCI *vs* controls; and an about 130% higher CXCL9 level, 13% lower fractalkine (CX3CL1) level, 37% higher CCL1 level in AD *vs* MCI. Meanwhile, in CSF, AD is linked to an around one-fifth higher level of CCL2 (MCP-1), and MCI to a modest rise (16%), when compared to healthy controls.

Chemokine-mediated neuroinflammation appears to play a significant role in the development and maintenance of cognitive impairment, according to an increasing body of evidence [15, 88, 89]. Chemokine liberation is high, which accelerates the inflammatory cascade. Chemokines are small proteins with 60–90 amino acids that exert an important function in directing leukocytes to areas of inflammation or injury during immune responses [14]. Some chemokines are thought to be pro-inflammatory and capable of inducing immune responses, whereas others are thought to be homeostatic.

CCL15, also known as macrophage inflammatory protein (MIP)-1δ, and CCL1 (I-309), belong to members of the CC chemokines. Both chemokines are important in attracting immune cells to sites of damage or infection. CCL1 is an atypical chemokine since it is released by more mobile T-lymphocytes, implying a broader immunological response. CCL1 level was observed to be higher in AD compared to controls and MCI in the transition from MCI to AD, regardless of age, sex, or APOE genotype, at each of the baseline, 18-, and 36-month sampling periods [60]. However, new data on CCL1 levels in CSF did not corroborate the finding that CCL1 in CSF, but not in blood, is linked with the severity of cognitive impairment [90]. As a macrophage inflammatory protein that binds to its receptor and exerts a pro-inflammatory effect [91, 92], CCL15 increased cell adhesion of monocytes to endothelial cells under static and shear-stress conditions [93]. Since their effect sizes are large, the blood levels of both the chemokines were useful in differentiating AD from MCI and healthy participants based on our meta-analysis.

CCL2, also called as MCP-1, is a CC chemokine that plays a key role in AD-related neuroinflammation [16]. CCL2 is a crucial component of the neuroinflammatory response that is produced by Aβ-stimulated microglia and astrocytes [94]. CCL2 loss was found to affect behavioral impairments and disease development in Aβ precursor protein/presenilin-1 double-transgenic mice [95, 96], implying that CCL2 signaling is important in AD [97]. CCL2 was found to be involved in the rupture of the blood–brain barrier in an acute neurological illness model [98]. Studies have reported that increased CSF MCP-1 levels are linked to lower MMSE scores, and greater baseline levels predict a faster rate of cognitive deterioration in the early stages of Alzheimer’s disease [24]. As a result, CCL2 could be used as a measure of AD progression [99]. Our meta-analytic findings revealed that MCP-1 levels were significantly elevated in CSF, but not in blood, in subjects with AD and MCI, suggesting that increased MCP-1 level appears to be primarily from CNS-resident cells rather than from peripheral leucocytes, and that the CSF levels of MCP-1 had a larger effect size in AD-controls than in MCI-controls, indicating that increased CSF MCP-1 level is clearly associated with the severity of cognitive impairment.

CCL27 is known as the cutaneous T cell attractive chemokine because it is predominantly produced by keratinocytes in the skin and has memory T cell homing capabilities (CTACK). It has a high level of expression in the central nervous system, particularly in the cerebral cortex and limbic structures [100], as well as in the liver and kidneys [101]. The chemokine CCL27 transcript was highly upregulated at the locations of AD lesions [102]. Blood CCL27 may be a good marker that can differentiate AD from healthy subjects, but more investigations on the relationship between AD and blood CCL27, as well as upon the role of CCL27 in Alzheimer’s neurodegeneration are urgently needed.

The chemokines CXCL9 and CXCL10 (IP-10) share CXCR3 as a common receptor, which is expressed on T cells, NK cells, and neurons. CXCL10 was found to be expressed in astrocytes and to be localized around Aβ plaques in an AD mouse model [103]. CXCL10 is upregulated in rat brains, cultured astrocytes, and microglia after LPS injection, indicating that it is implicated in inflammatory processes. In an APP/PS1 mouse model, CXCR3 deletion significantly reduced plaque formation in the brain [103, 104]. In current analyses, although having very less number of studies, both the chemokines were found to have large effect sizes ranging from 1.78, 1.92, and 2.29. As a result, our meta-analysis revealed that CXCL9 and CXCL10 might also be useful as tau-independent and Aβ-independent blood-based candidate biomarkers for AD.

In nervous system, the chemokine CXCL8 (IL-8) is expressed in neurons, astrocytes, and microglia. Its receptor CXCR2 is highly expressed in microglia and astrocytes. When in vitro stimulated with Aβ, microglia, astrocytes, and neurons were all capable of producing CXCL8. It was reported that IL-8 could affect GSK3β phosphorylation and modulate protein phosphatase activity in vitro, resulting in enhanced Tau phosphorylation [105]. A study [106] found that CXCL8 levels in AD brain were considerably greater than those in age-matched controls. As a result, it may play a detrimental role in the etiology of Alzheimer’s disease. The current analysis has demonstrated that blood IL-8 has a large effect size, a strong significance in the comparison of AD with healthy individuals, if the outlier was removed, meaning that more investigations is needed to clarify this.

The only member of the CX3C family, CX3CL1 (fractalkine), is one of only two transmembrane chemokines. It is found in neurons, astrocytes, and endothelial cells; the fractalkine-specific receptor, G protein-coupled CX3CR1, is expressed in astrocytes and microglia, and the CX3CL1-CX3CR1 interaction controls microglial recruitment to neuroinflammation sites. Neuronal survival, plaque load, and cognition are all influenced by the CX3CR1/CX3CL1 system [107]. Fractalkine expression in the hippocampus and cortex is lower in AD than in non-demented controls [108], indicating that this CX3CL1-CX3CR1 pathway is dysregulated in AD. It may have complex interactions with the two characteristic hallmarks of AD and may be neuroprotective [109, 110] or neurotoxic [89] at various stages of disease progression. Although the blood fractalkine ratio is not statistically significant between AD and controls in the current study, the considerable large effect size in MCI *vs* control does not rule out its potential as a biomarker for separating MCI patients from healthy subjects. Meanwhile, our finding would seem to hint that there was a fluctuating change in the blood fractalkine levels during cognitive impairment, with stages of a rapid rise in modest impairment, then an obvious drop in serious impairment.

This meta-analysis differed from previous reports [34, 111] in several important aspects. First, it was larger and more comprehensive than ever. Second, we have applied the approach of generating fold-change using the ratio of means (RoM) as the effect measure to control the variability in concentration ranges between studies. The variability in chemokine concentrations between laboratories and assays, as well as in varied cutpoints, is high; however, the RoM, as a measure superior to standard mean difference, can help reduce this. Third, those studies in control individuals diagnosed with depression, headache, or pain syndromes were excluded, which would reduce any potential impact of common clinical complaints on chemokine concentrations.

Some limitations should be addressed in this analysis. Firstly, in most cohorts, a significant problem is a lesser number of studies in a single chemokine, and there was noticeable heterogeneity. Secondly, although the exclusion of depression or headache or pain from HC groups may indeed result in the selection of healthier comparator groups, this generates its own problems in that it is not possible to know whether the measured chemokine levels are specific to AD/MCI. Comparisons would have been enhanced by including groups with other brain disease, e.g., Parkinson’s disease or stroke. Thirdly, most of the studies included were case–control designs in the review—these are likely to enter significant bias. Fourthly, most of the studies included failed to either exclude or describe the use of anti-inflammatory drugs, which can substantially affect the levels of chemokines. Lastly, it is likely that a few of these chemokines are correlated with age. Thus, there is the possibility that the changes in AD or MCI are driven by age, particularly if the AD or MCI patients are older than HC.

Some chemokines, such as CCL19, revealed changes in concentrations between AD and controls, but were unable to be distinguished using ratios. Among most of the comparisons, there were a limited number of studies. As a result, our findings should be regarded only as exploratory and hypothesis-generating. However, the reported overall effect sizes of blood marker performance provide useful information for future research. Furthermore, the finding of the pivotal chemokines linked to AD and MCI has their potential to remove hurdles of therapeutic development. Meanwhile, more research is needed to determine how these peripheral or CSF chemokines are linked to well-established AD biomarkers like Aβ and tau.

## Conclusions

Our meta-analysis revealed significant relationships of blood CXCL10, CXCL9, CCL27, and CCL15, as well as CSF CCL2 with Alzheimer’s patients compared with cognitively normal control subjects, of blood CXCL9 and CCL1 with Alzheimer’s disease compared with mild cognitive impairment, and of blood CX3CL1 with mild cognitive impairment compared with healthy subjects. CCL2 (MCP-1) may be the only CSF chemokine biomarker for the comparisons of AD or MCI with healthy people. However, these findings must be verified in future large and multicenter cohort studies for subsequent diagnosis and/or prognostic utility for MCI and AD.

## Supplementary Information

**Additional file 1: Table S1.** Search strategy in this systematic review and meta-analysis. **Table S2.** List of excluded studies and reason for exclusion. **Figure S1.** Sensitivity analyses for Alzheimer’s disease to healthy control ratio of mean serum/plasma chemokine CXCL8 (IL-8). **Figure S2.** Funnel plots of blood chemokine CCL1 in AD *vs* HC. **Figure S3.** Funnel plots of blood chemokines CCL2, CCL3, CCL4, CCL5 in AD *vs* HC. **Figure S4.** Funnel plots of blood chemokines CCL7, CCL11, CCL15, CCL17 in AD *vs *HC. **Figure S5.** Funnel plots of blood chemokines CCL26, CCL27, CXCL1, CXCL8 in AD *vs* HC. **Figure S6.** Funnel plots of blood chemokines CXCL9, CXCL10, CXCL12 and CX3CL1 in AD *vs* HC. **Figure S7.** Funnel plots of blood chemokines CCL2, CCL4, CCL11, CXCL8 in MCI *vs* HC. **Figure S8.** Funnel plots of blood chemokines CX3CL1 in MCI vs HC. **Figure S9.** Funnel plots of blood chemokines CCL2, CCL4, CXCL8 in AD *vs* MCI. **Figure S10.** Funnel plots of CSF chemokines CCL2, CXCL8, CXCL10 in AD *vs* HC. **Figure S11.** Funnel plots of CSF chemokines CCL2, CXCL8, CXCL10, CX3CL1 in MCI *vs* HC. **Figure S12.** Funnel plots of AD to MCI ratio of mean for CSF chemokines CCL2, CXCL8, CX3CL1. **Figure S13.** Forest plots of RoM for AD/HC in serum/plasma chemokine levels. **Figure S14.** Forest plots of RoM for MCI/HC and AD/MCI in serum/plasma chemokine levels. **Figure S15.** Forest plots of RoM for AD/HC, MCI/HC, and AD/MCI in CSF chemokine levels. **Figure S16.** Subgroup analyses of RoM for AD to HC in blood/CSF chemokine CCL2 (MCP-1) levels. **Figure S17.** Subgroup analyses of RoM for AD to HC/MCI in blood/CSF chemokine CCL2 (MCP-1) levels. **Figure S18.** Subgroup analyses of RoM for MCI to HC in blood/CSF chemokine CCL2 (MCP-1) levels. **Figure S19.** Subgroup analyses of RoM for AD to HC in blood chemokine CXCL8 (IL-8) levels. **Figure S20.** Subgroup analyses of RoM for MCI to HC in blood/CSF chemokine CXCL8 (IL-8) levels.

## Data Availability

Not applicable.
