# Supplementary material for: Blood and CSF chemokines in Alzheimer’s disease and mild cognitive impairment: a systematic review and meta-analysis
Source: Alzheimers Res Ther. 2023 Jun 8;15:107. doi: 10.1186/s13195-023-01254-1 (PMC10249313; doi:10.1186/s13195-023-01254-1)
Supplement: Supplementary file 1 — Additional file 1: Table S1. Search strategy in this systematic review and meta-analysis. Table S2. List of excluded studies and reason for exclusion. Figure S1. Sensitivity analyses for Alzheimer’s disease to healthy control ratio of mean serum/plasma chemokine CXCL8 (IL-8). Figure S2. Funnel plots of blood chemokine CCL1 in AD vs HC. Figure S3. Funnel plots of blood chemokines CCL2, CCL3, CCL4, CCL5 in AD vs HC. Figure S4. Funnel plots of blood chemokines CCL7, CCL11, CCL15, CCL17 in AD vs HC. Figure S5. Funnel plots of blood chemokines CCL26, CCL27, CXCL1, CXCL8 in AD vs HC. Figure S6. Funnel plots of blood chemokines CXCL9, CXCL10, CXCL12 and CX3CL1 in AD vs HC. Figure S7. Funnel plots of blood chemokines CCL2, CCL4, CCL11, CXCL8 in MCI vs HC. Figure S8. Funnel plots of blood chemokines CX3CL1 in MCI vs HC. Figure S9. Funnel plots of blood chemokines CCL2, CCL4, CXCL8 in AD vs MCI. Figure S10. Funnel plots of CSF chemokines CCL2, CXCL8, CXCL10 in AD vs HC. Figure S11. Funnel plots of CSF chemokines CCL2, CXCL8, CXCL10, CX3CL1 in MCI vs HC. Figure S12. Funnel plots of AD to MCI ratio of mean for CSF chemokines CCL2, CXCL8, CX3CL1. Figure S13. Forest plots of RoM for AD/HC in serum/plasma chemokine levels. Figure S14. Forest plots of RoM for MCI/HC and AD/MCI in serum/plasma chemokine levels. Figure S15. Forest plots of RoM for AD/HC, MCI/HC, and AD/MCI in CSF chemokine levels. Figure S16. Subgroup analyses of RoM for AD to HC in blood/CSF chemokine CCL2 (MCP-1) levels. Figure S17. Subgroup analyses of RoM for AD to HC/MCI in blood/CSF chemokine CCL2 (MCP-1) levels. Figure S18. Subgroup analyses of RoM for MCI to HC in blood/CSF chemokine CCL2 (MCP-1) levels. Figure S19. Subgroup analyses of RoM for AD to HC in blood chemokine CXCL8 (IL-8) levels. Figure S20. Subgroup analyses of RoM for MCI to HC in blood/CSF chemokine CXCL8 (IL-8) levels. [file 13195_2023_1254_MOESM1_ESM.docx]

**Supplemental material**

**Figure and table legends**

**Table S1. Search strategy in this systematic review and meta-analysis.**

**Table S2. List of excluded studies and reason for exclusion**

**Figure S1: Sensitivity analyses for Alzheimer’s disease to healthy control ratio of mean serum/plasma chemokine CXCL8 (IL-8).**

**Figure S2. Funnel plots of blood chemokine CCL1 in AD *vs* HC.**

**Figure S3. Funnel plots of blood chemokines CCL2, CCL3, CCL4, CCL5 in AD *vs* HC.**

**Figure S4. Funnel plots of blood chemokines CCL7, CCL11, CCL15, CCL17 in AD *vs* HC.**

**Figure S5. Funnel plots of blood chemokines CCL26, CCL27, CXCL1, CXCL8 in AD *vs* HC.**

**Figure S6. Funnel plots of blood chemokines CXCL9, CXCL10, CXCL12 and CX3CL1 in AD *vs* HC.**

**Figure S7. Funnel plots of blood chemokines CCL2, CCL4, CCL11, CXCL8 in MCI *vs* HC.**

**Figure S8. Funnel plots of blood chemokines CX3CL1 in MCI vs HC.**

**Figure S9. Funnel plots of blood chemokines CCL2, CCL4, CXCL8 in AD *vs* MCI .**

**Figure S10. Funnel plots of CSF chemokines CCL2, CXCL8, CXCL10 in AD *vs* HC.**

**Figure S11. Funnel plots of CSF chemokines CCL2, CXCL8, CXCL10, CX3CL1 in MCI *vs* HC.**

**Figure S12. Funnel plots of AD to MCI ratio of mean for CSF chemokines CCL2, CXCL8, CX3CL1.**

**Figure S13: Forest plots of RoM for AD/HC in serum/plasma chemokine levels.**

**Figure S14: Forest plots of RoM for MCI/HC and AD/MCI in serum/plasma chemokine levels.**

**Figure S15: Forest plots of RoM for AD/HC, MCI/HC, and AD/MCI in CSF chemokine levels.**

**Figure S16. Subgroup analyses of RoM for AD to HC in blood/CSF chemokine CCL2 (MCP-1) levels.**

**Figure S17. Subgroup analyses of RoM for AD to HC/MCI in blood/CSF chemokine CCL2 (MCP-1) levels.**

**Figure S18. Subgroup analyses of RoM for MCI to HC in blood/CSF chemokine CCL2 (MCP-1) levels.**

**Figure S19. Subgroup analyses of RoM for AD to HC in blood chemokine CXCL8 (IL-8) levels.**

**Figure S20. Subgroup analyses of RoM for MCI to HC in blood/CSF chemokine CXCL8 (IL-8) levels.**

**Table S1. Search strategy in this systematic review and meta-analysis.**

| Search | Query |
| --- | --- |
| 1 | chemokine*[tiab] |
| 2 | chemokine[tiab] |
| 3 | ccl1[tiab] or ccl2[tiab] or ccl3[tiab] or ccl4[tiab] or ccl5[tiab] or ccl6[tiab] or ccl7[tiab] or ccl8[tiab] or ccl9[tiab] or ccl10[tiab] or ccl11[tiab] or ccl12[tiab] or ccl13[tiab] or ccl14[tiab] or ccl15[tiab] or ccl16[tiab] or ccl17[tiab] or ccl18[tiab] or ccl19[tiab] or ccl20[tiab] or ccl21[tiab] or ccl22[tiab] or ccl23[tiab] or ccl24[tiab] or ccl25[tiab] or ccl26[tiab] or ccl27[tiab] or ccl28[tiab] or ccl[tiab] |
| 4 | cxcl1[tiab] or cxcl2[tiab] or cxcl3[tiab] or cxcl4[tiab] or cxcl5[tiab] or cxcl6[tiab] or cxcl7[tiab] or cxcl8[tiab] or cxcl9[tiab] or cxcl10[tiab] or cxcl11[tiab] or cxcl12[tiab] or cxcl13[tiab] or cxcl14[tiab] or cxcl15[tiab] or cxcl16[tiab] or cxcl17[tiab] or cxcl18[tiab] or cxcl19[tiab] or cxcl20[tiab] or cxcl21[tiab] or cxcl[tiab] |
| 5 | xcl1[tiab] or xcl2[tiab] or xcl[tiab] |
| 6 | cx3cl1[tiab] or cx3[tiab] or cx3cl[tiab] |
| 7 | scya1[tiab] or scya2[tiab] or scya3[tiab] or scya4[tiab] or scya5[tiab] or scya6[tiab] or scya7[tiab] or scya8[tiab] or scya9[tiab] or scya10[tiab] or scya11[tiab] or scya12[tiab] or scya13[tiab] or scya14[tiab] or scya15[tiab] or scya16[tiab] or scya17[tiab] or scya18[tiab] or scya19[tiab] or scya20[tiab] or scya21[tiab] or scya22[tiab] or scya23[tiab] or scya24[tiab] or scya25[tiab] or scya26[tiab] or scya[tiab] |
|  | scyb1[tiab] or scyb2[tiab] or scyb3[tiab] or scyb4[tiab] or scyb5[tiab] or scyb6[tiab] or scyb7[tiab] or scyb8[tiab] or scyb9[tiab] or scyb10[tiab] or scyb11[tiab] or scyb12[tiab] or scyb13[tiab] or scyb14[tiab] or scyb[tiab] |
| 8 | Scyc1[tiab] or scyc[tiab] |
| 9 | Scyd1[tiab] or scyd[tiab] |
| 10 | "small inducible cytokine"[tiab] |
| 11 | ccr1[tiab] or ccr2[tiab] or ccr2b[tiab] or ccr3[tiab] or ccr4[tiab] or ccr5[tiab] or ccr6[tiab] or ccr7[tiab] or ccr8[tiab] or ccr9[tiab] or ccr10[tiab] or ccr11[tiab] or ccr12[tiab] or ccr13[tiab] or ccr[tiab] |
| 12 | cxcr1[tiab] or cxcr2[tiab] or cxcr3[tiab] or cxcr3b[tiab] or cxcr4[tiab] or cxcr5[tiab] or cxcr6[tiab] or cxcr7[tiab] or cxcr8[tiab] or cxcr9[tiab] or cxcr10[tiab] or cxcr[tiab] |
| 13 | xcr1[tiab] or xcr[tiab] |
| 14 | cx3cr1[tiab] or cx3cr[tiab] or cx3[tiab] |
| 15 | chemotactic[tiab] |
| 16 | i-309[tiab] or i309[tiab] or tca[tiab] or tca3[tiab] or sise[tiab] |
| 17 | Mcp[tiab] or mcp1[tiab] |
| 18 | Mip1[tiab] or "macrophage inflammatory protein"[tiab] or mip-1[tiab] |
| 19 | rantes[tiab] or "regulated on activation, normal t cell expressed and secreted"[tiab] |
| 20 | mrp[tiab] |
| 21 | eotaxin[tiab] or eotaxin1 or eotaxin2 or eotaxin3 |
| 22 | leukotactin[tiab] |
| 23 | 6ckine[tiab] |
| 24 | gro[tiab] |
| 25 | "platelet factor"[tiab] |
| 26 | "myeloid progenitor inhibitory factor"[tiab] |
| 27 | chemoattractant[tiab] |
| 28 | "interleukin 8"[tiab] or IL-8[tiab] or IL8 |
| 29 | lungkine[tiab] |
| 30 | fractalkine[tiab] |
| 31 | neurotactin[tiab] |
| 32 | lymphotactin[tiab] |
| 33 | IP10[tiab] or IP-10[tiab] |
| 34 | 1 OR 2 OR 3 OR 4 OR 5 OR 6 OR 7 OR 8 OR 9 OR 10 OR 11 OR 12 OR 13 OR 14 OR 15 OR 16 OR 17 OR 18 OR 19 OR 20 OR 21 OR 22 OR 23 OR 24 OR 25 OR 26 OR 27 OR 28 OR 29 OR 30 OR 31 OR 32 OR 33 |
| 35 | "Alzheimer’s disease"[tiab] OR Alzheimer[tiab] OR Alzheimer* OR "Alzheimer’s"[tiab] OR "cognitive impairment"[tiab] OR "MCI" [tiab] OR cognit*[tiab] OR dementia*[tiab] OR demented[tiab] |
| 36 | 34 AND 35 |
| 37 | 36 not review[pt] not meta-analysis[ti] |

**Table S2. List of excluded studies and reason for exclusion**

| **Exclusion reason** | **Reference number** |
| --- | --- |
| Unrelated topic | [1-9] |
| Non-plasma/serum or CSF source, e.g. brain tissue | [10-26], [27-37] |
| No comparison between cases and healthy controls | [38-47], [48-57], [58-66] |
| Grouping not based on AD or MCI | [67-79], [80-87] |
| Measures (mean/SD/N) unavailable | [88-109], [110-119] |
| Review type | [120, 121] |
| Replicate or not independent study | [122-125] |
| Neither English nor Chinese languages | [126] |
| Only one study in the CSF level of a single chemokine | [127], [128] |

**Figure S1. Funnel plots of blood chemokine CCL1 in AD *vs* HC.**


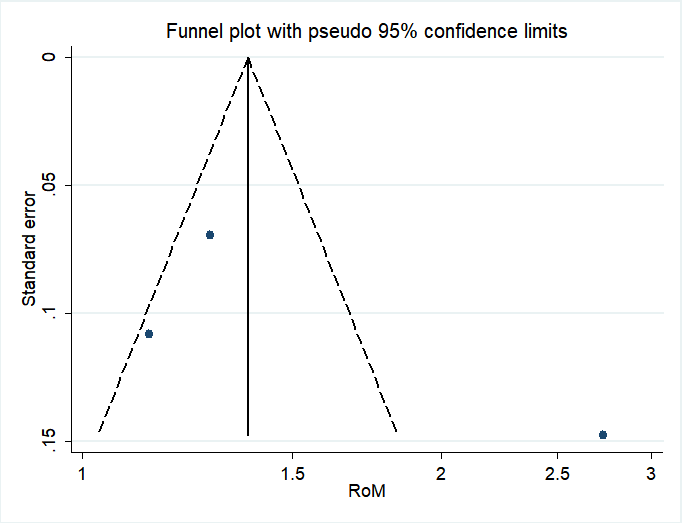


**Figure S2: Sensitivity analyses for Alzheimer’s disease to healthy control ratio of mean serum/plasma chemokine CXCL8 (IL-8).**


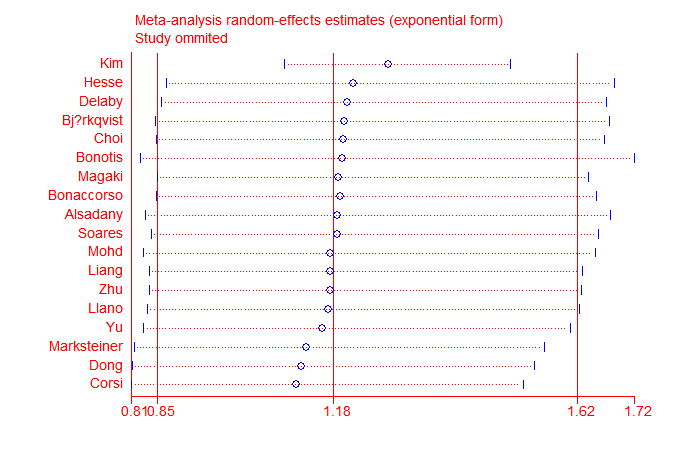


**Figure S3. Funnel plots of blood chemokines CCL2 (A), CCL3 (B), CCL4 (C), CCL5 (D) in AD *vs* HC.**

**A B**

Begg’s test: p=0.02, Egger’s test: p=0.06 in CCL2; Begg’s test: p=1, Egger’s test: p=0.72 in CCL3

adjusted RoM (95% CI): 1.13 (0.92-1.39)

using the trim-and-filled method.

**C D**

Begg’s test: p=0.31, Egger’s test: p=0.09 in CCL4. Begg’s test: p=1, Egger’s test: p=0.57 in CCL5

**Figure S4. Funnel plots of blood chemokines CCL7 (A), CCL11 (B), CCL15 (C), CCL17 (D) in AD *vs* HC.**

**A B**

Begg’s test: p=1, Egger’s test: p=0.76 in CCL7. Begg’s test: p=1, Egger’s test: p=0.80 in CCL11.

**C D**


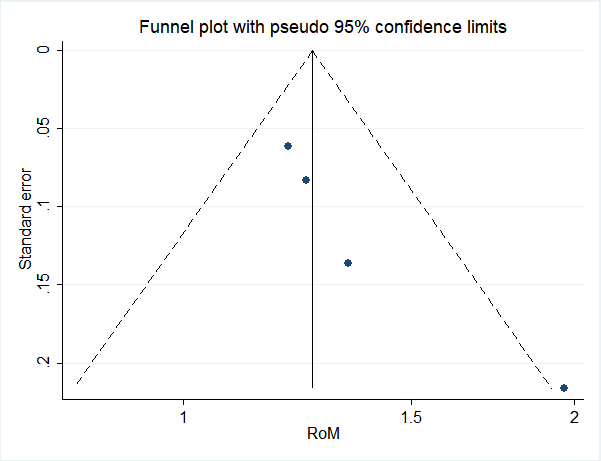


Begg’s test: p=0.09, Egger’s test: p=0.06 in CCL15. Begg’s test: p=0.31, Egger’s test: p=0.60 in CCL17

**Figure S5. Funnel plots of blood chemokines CCL26 (A), CCL27 (B), CXCL1 (C), CXCL8 (D) in AD *vs* HC.**

**A B**

Begg’s test: p=1, Egger’s test: p=0.25 in CCL26 Begg’s test: p=0.30, Egger’s test: p=0.55 in CCL27

**C D**

Begg’s test: p=0.31, Egger’s test: p=0.41 in CXCL1. Begg’s test: p=0.77, Egger’s test: p=0.21 in CXCL8 with the exception of the outlier (kim et al.’s study [129]).

**Figure S6. Funnel plots of blood chemokines CXCL9 (A), CXCL10 (B), CXCL12 (C) and CX3CL1 (D) in AD *vs* HC.**

**A B**


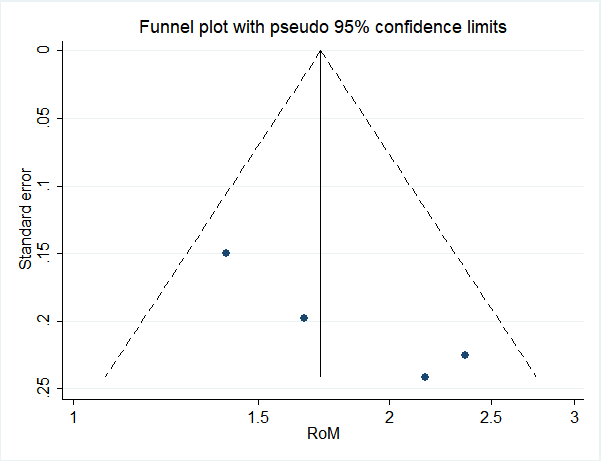

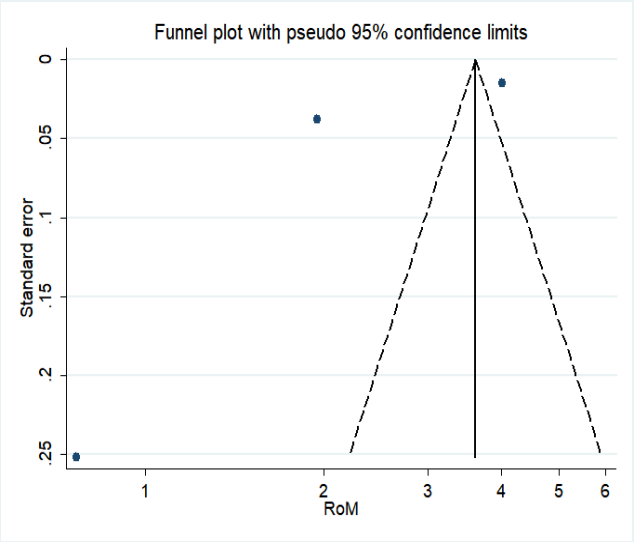


Begg’s test: p=0.31 Egger’s test: p=0.05 in CXCL9 Begg’s test: p=1, Egger’s test: p=0.44 in CXCL10

**C D**

Begg’s test: p=1, Egger’s test: p=0.97 in CXCL12 Begg’s test: p=1, Egger’s test: p=0.51 in CX3CL1

**Figure S7. Funnel plots of blood chemokines CCL2 (A), CCL4 (B), CCL11 (C), CXCL8 (D) in MCI *vs* HC.**

**A B**

Begg’s test: p=0.50, Egger’s test: p=0.412 in CCL2. Begg’s test: p=1, Egger’s test: p=0.51 in CCL4

**C D**

Begg’s test: p=1, Egger’s test: p=0.49 in CCL11. Begg’s test: p=0.45, Egger’s test: p=0.79 in CXCL8 with the exception of the outlier (Kim et al.’s study [129]) .

**Figure S8. Funnel plots of blood chemokine CX3CL1 in MCI vs HC.**

Begg’s test: p=1, Egger’s test: p=0.63 in CX3CL1

**Figure S9. Funnel plots of blood chemokines CCL2 (A), CCL4 (B), CXCL8 (C) in AD *vs* MCI .**

**A B**

Begg’s test: p=0.60, Egger’s test: p=0.53 in CCL2 Begg’s test: p=0.31, Egger’s test: p=0.11 in CCL4

**C**


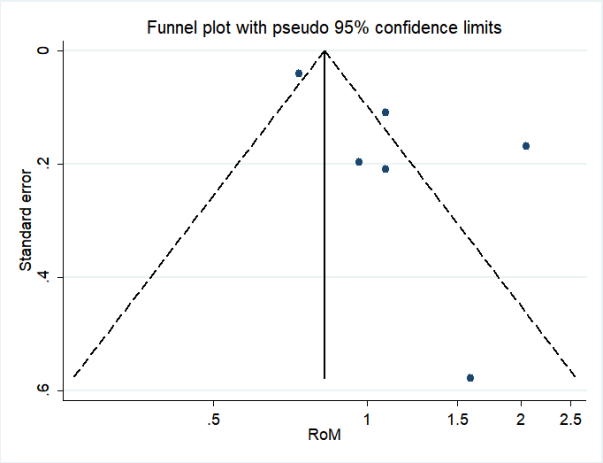


Begg’s test: p=1, Egger’s test: p=0.08 in CXCL8

**Figure S10. Funnel plots of CSF chemokines CCL2 (A), CXCL8 (B), CXCL10 (C) in AD *vs* HC.**

**A B**


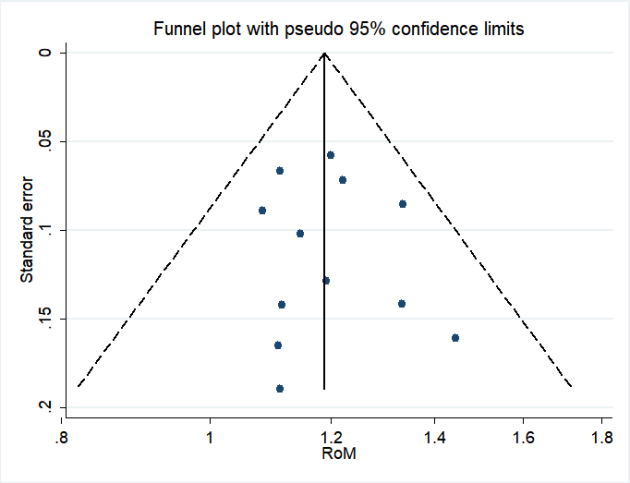


Begg’s test: p=0.84, Egger’s test: p=0.73 in CCL2. Begg’s test: p=0.59, Egger’s test: p=0.85 in CXCL8

**C D**

Begg’s test: p=0.46, Egger’s test: p=0.60 in CXCL10. Begg’s test: p=0.73, Egger’s test: p=0.71 in CX3CL1

**Figure S11. Funnel plots of CSF chemokines CCL2 (A), CXCL8 (B), CXCL10 (C), CX3CL1 (D) in MCI *vs* HC.**

**A B**

Begg’s test: p=0.26, Egger’s test: p=0.39 in CCL2 Begg’s test: p=1, Egger’s test: p=0.37 in CXCL8

**C**


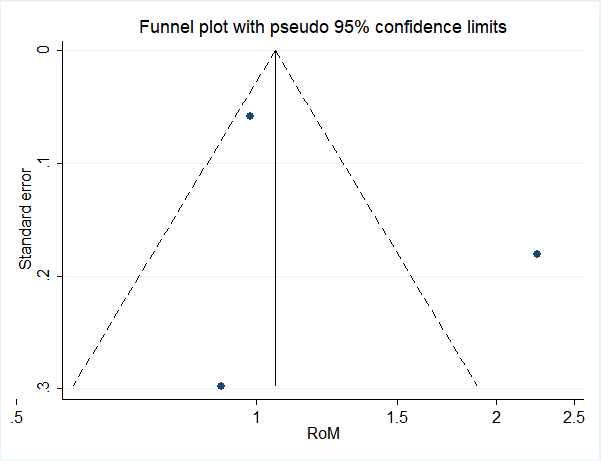


Begg’s test: p=1, Egger’s test: p=0.63 in CX3CL1

**Figure S12. Funnel plots of AD to MCI ratio of mean for CSF chemokines CCL2 (A), CXCL8 (B), CX3CL1 (C).**

**A**

Begg’s test: p=1, Egger’s test: p=0.33 in CX3CL1

**B C**

Begg’s test: p=1, Egger’s test: p=0.47 in CXCL8 Begg’s test: p=1, Egger’s test: p=0.33 in CCL2

**Figure S13: Forest plots of RoM for AD/HC in serum/plasma chemokine levels.**


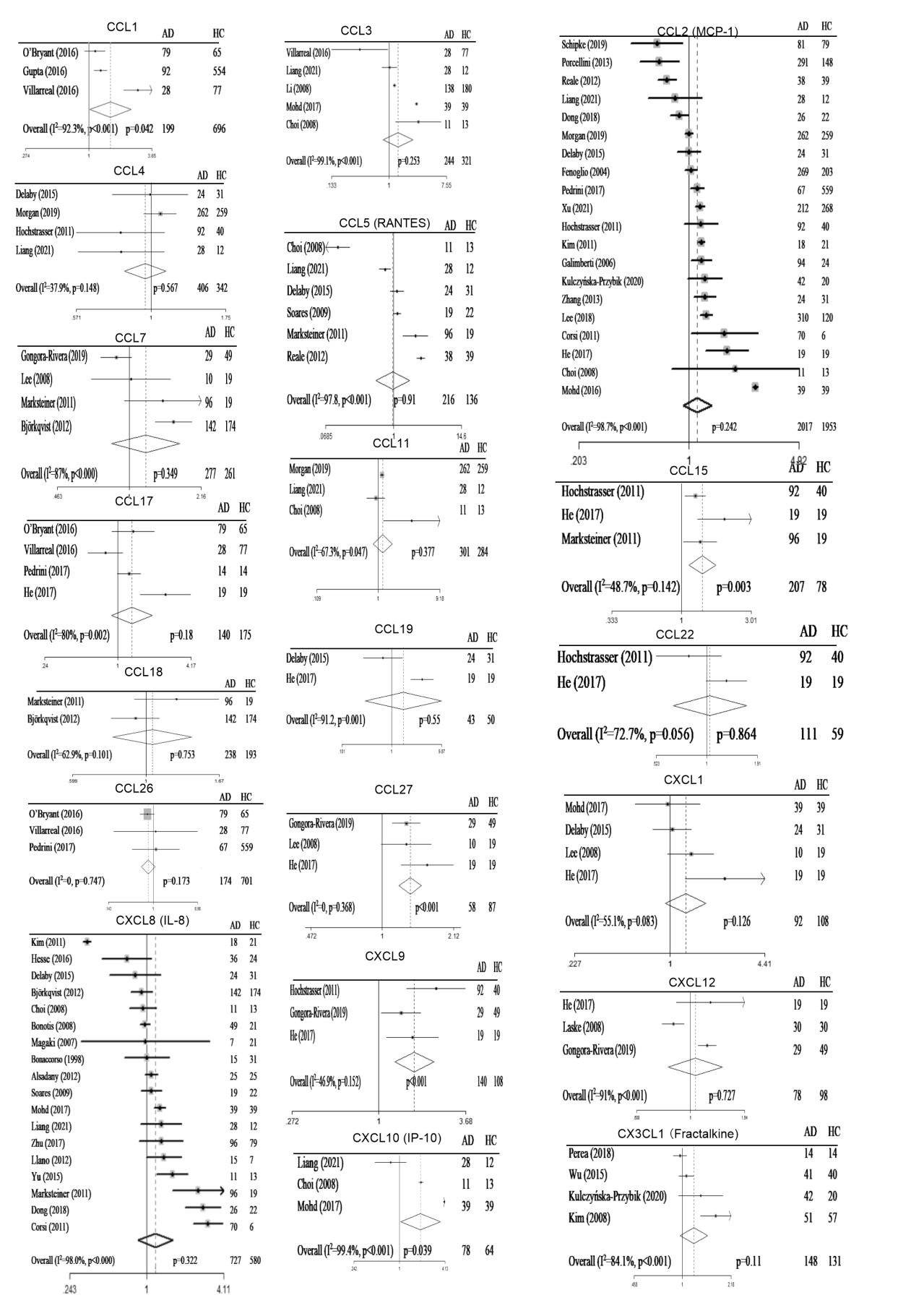


**Figure S14: Forest plots of RoM for MCI/HC and AD/MCI in serum/plasma chemokine levels.**

**
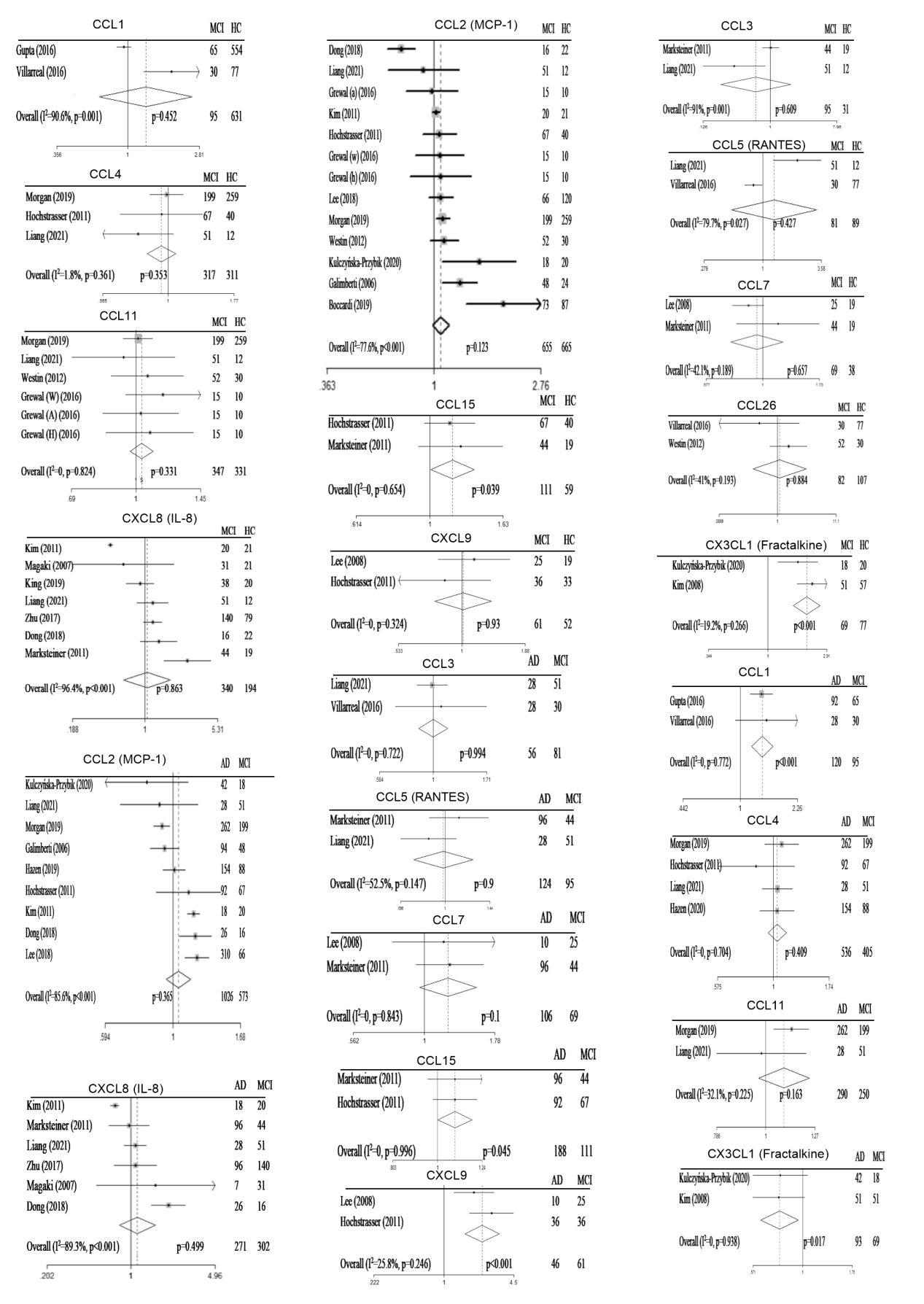
**

**Figure S15: Forest plots of RoM for AD/HC, MCI/HC, and AD/MCI in CSF chemokine levels.**


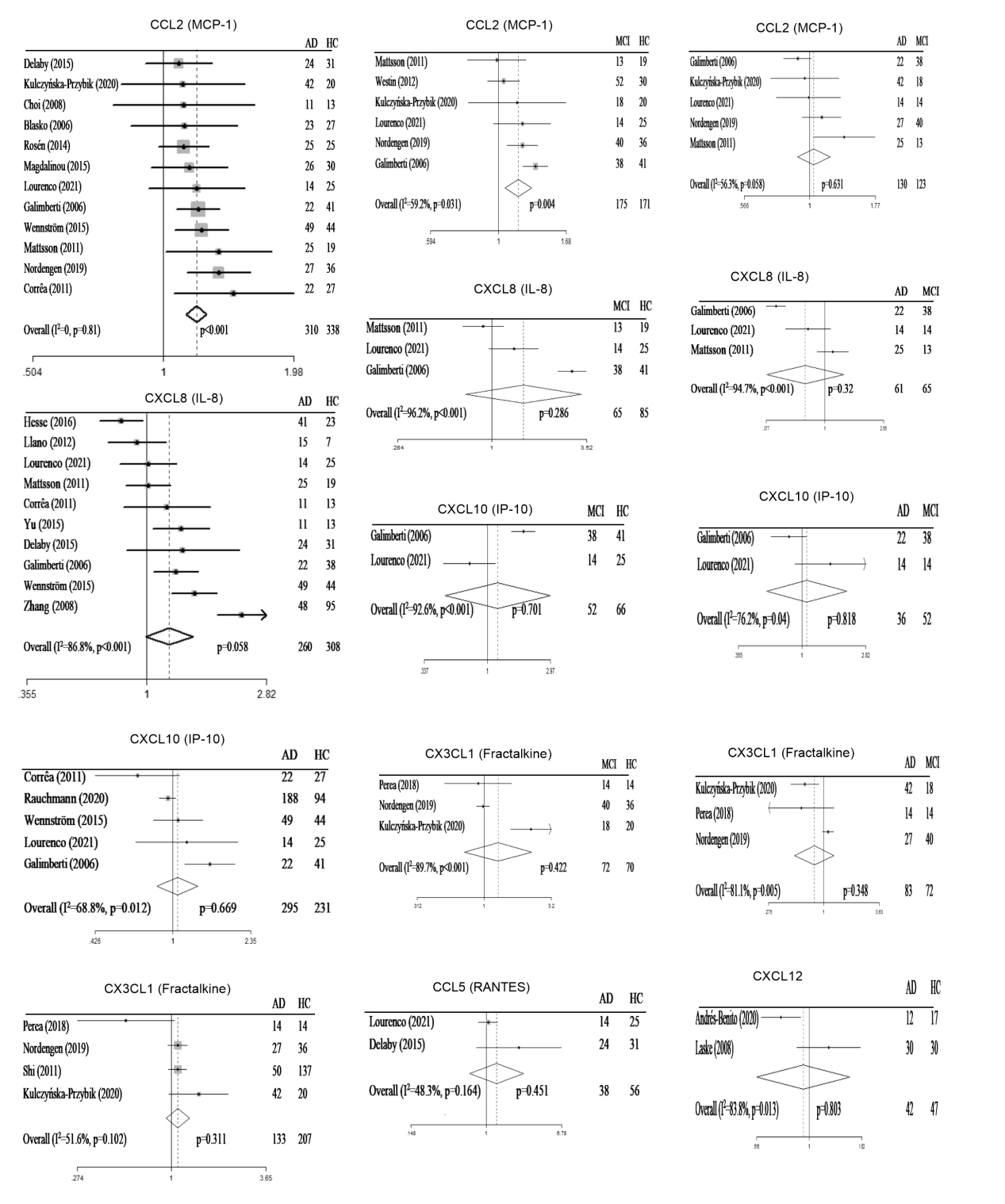


**Figure S16. Subgroup analyses of RoM for AD to HC in blood/CSF chemokine CCL2 (MCP-1) levels.**


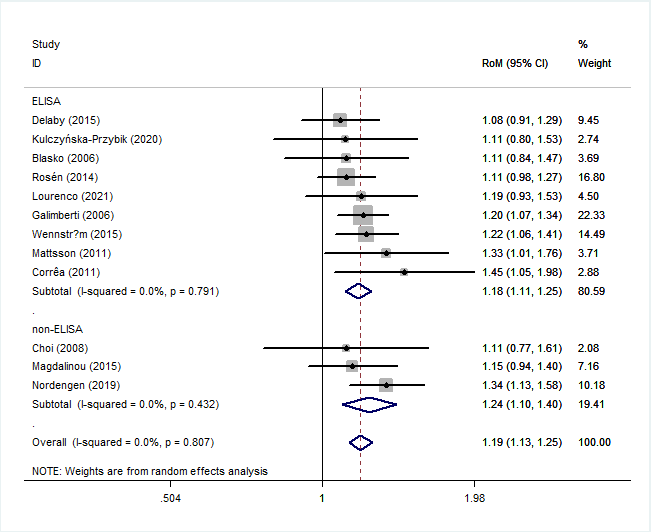

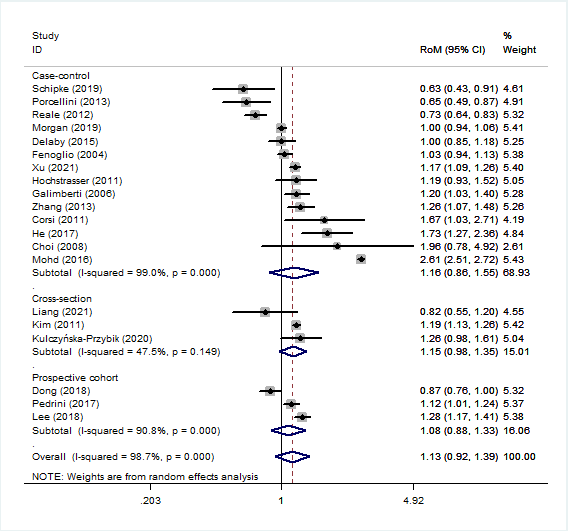


By assay method in CSF. By study design in blood.


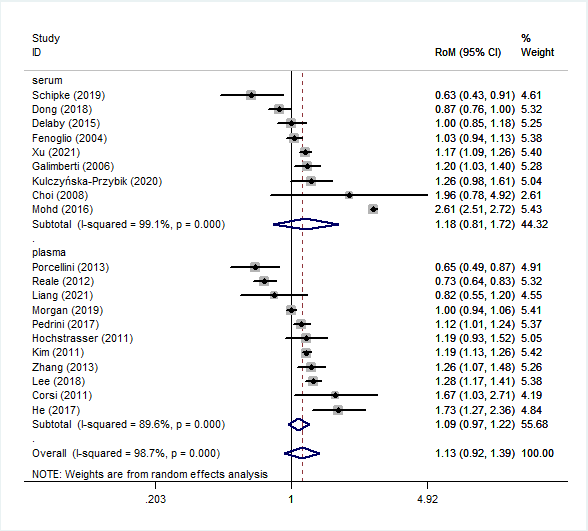

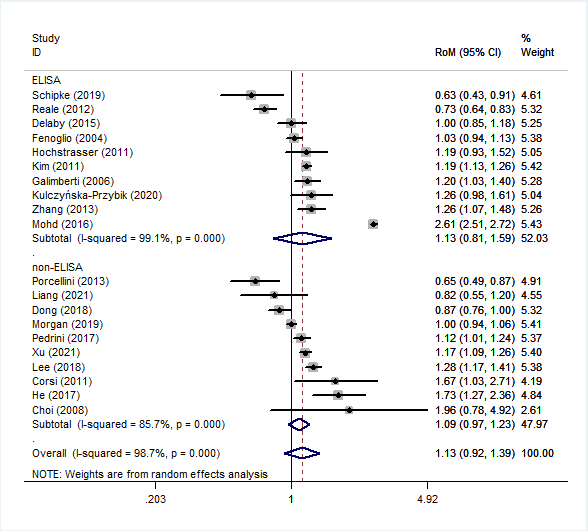


By sample source (serum/plasma). By assay method in blood.

**Figure S17. Subgroup analyses of RoM for AD to HC/MCI in blood/CSF chemokine CCL2 (MCP-1) levels.**


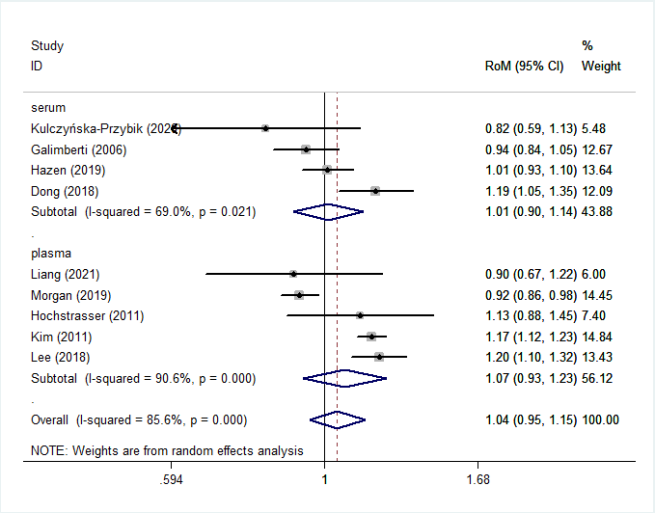

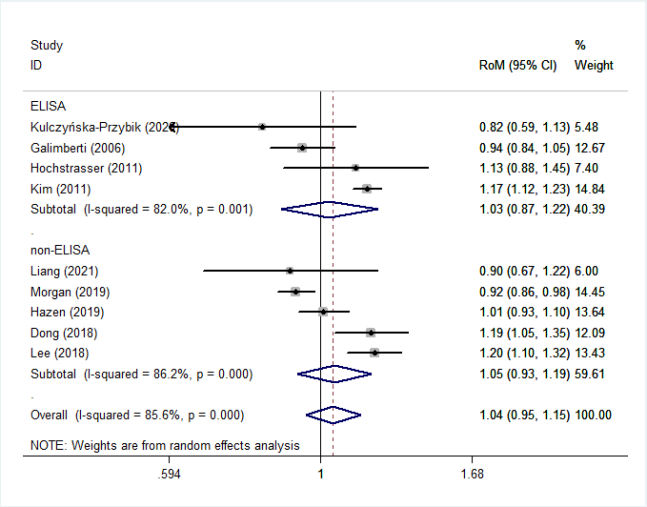


For AD to MCI by sample source. For AD to MCI by assay method in blood.


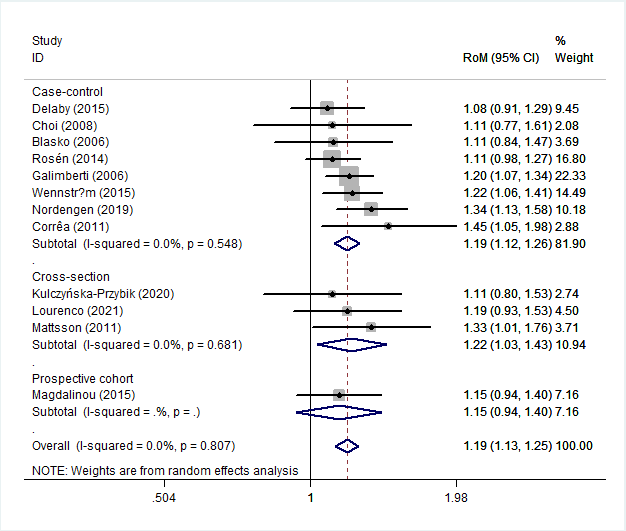


For AD to HC by study design in CSF.

**Figure S18. Subgroup analyses of RoM for MCI to HC in blood/CSF chemokine CCL2 (MCP-1) levels.**


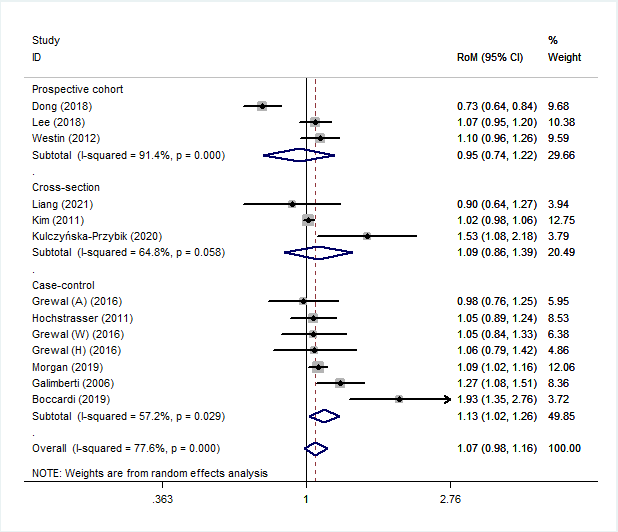

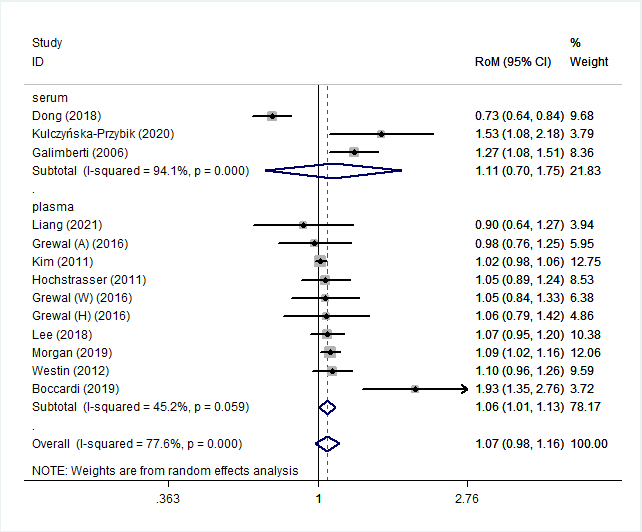


By study design in blood. By sample source (serum/plasma).


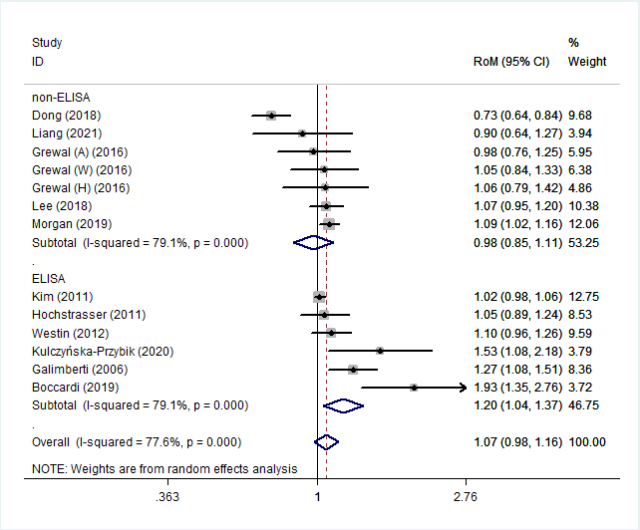

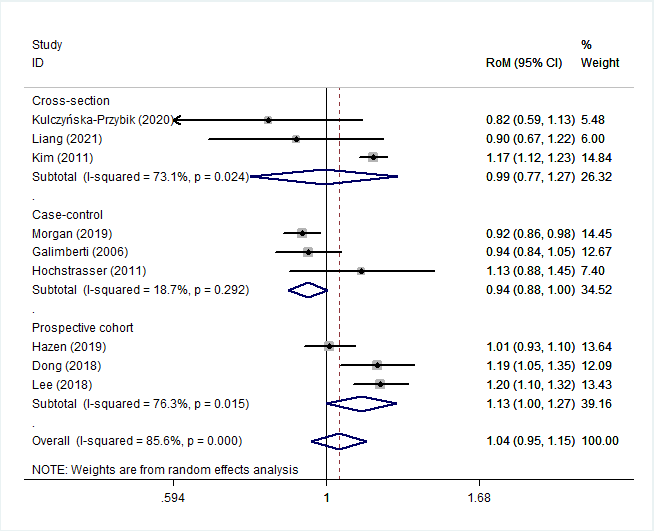


By assay method in blood. By study design in CSF.

**Figure S19. Subgroup analyses of RoM for AD to HC in blood chemokine CXCL8 (IL-8) levels.**


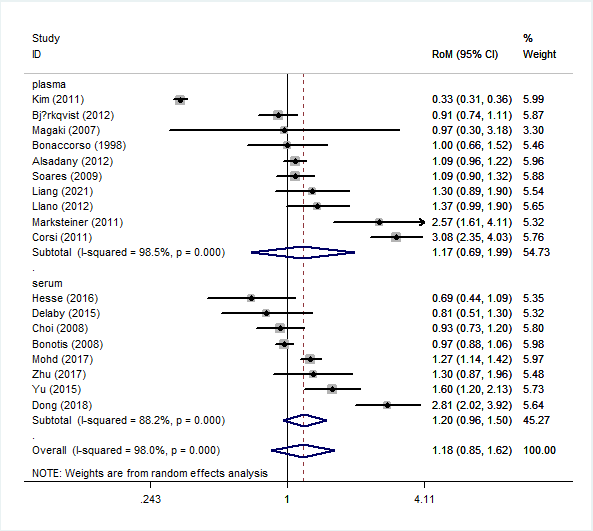


By sample source (serum/plasma).


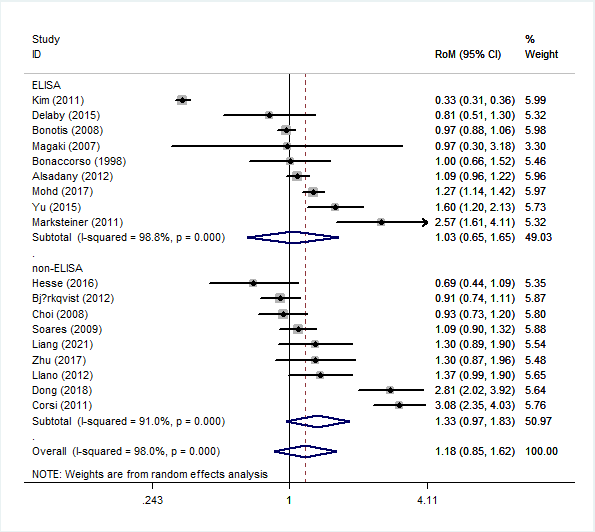


By assay method in blood.

**Figure S20. Subgroup analyses of RoM for MCI to HC in blood/CSF chemokine CXCL8 (IL-8) levels.**


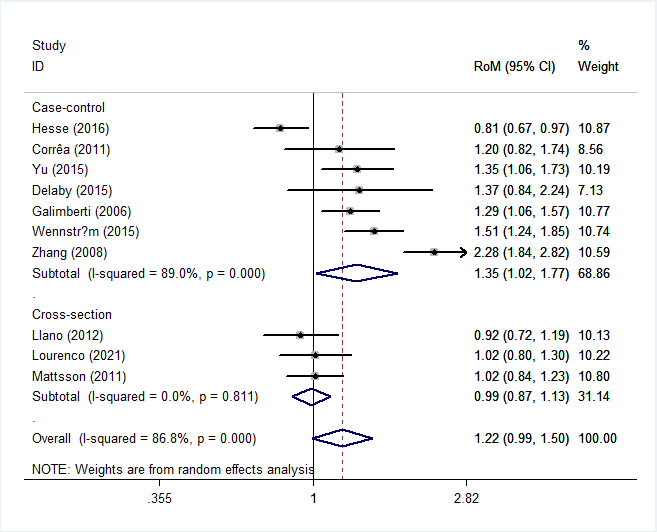


By study design in CSF.


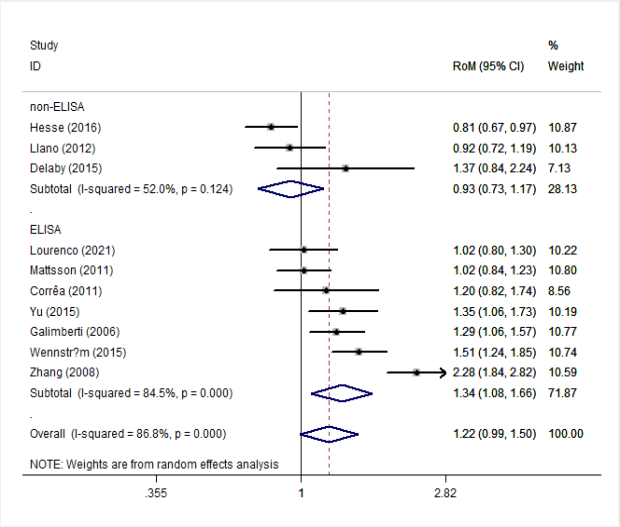

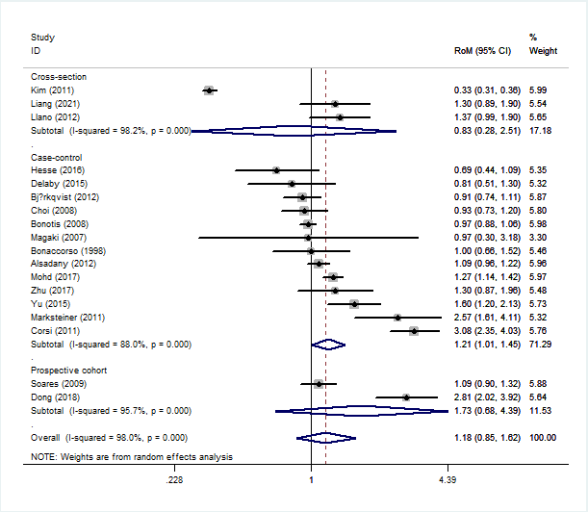


By assay method in CSF. By study design in blood.

**References**

[1] Sevush S, Jy W, Horstman LL, Mao WW, et al. Platelet activation in Alzheimer disease. Arch Neurol 1998, **55**: 530-536.

[2] Mohamed I, Ghanim H, Dandona P. Increased expression of alzheimer's disease related genes in obesity. Diabetes 2010.

[3] Chandrasekaran S, Bonchev D. Network Topology Analysis of Post-Mortem Brain Microarrays Identifies More Alzheimer's Related Genes and MicroRNAs and Points to Novel Routes for Fighting with the Disease. Plos One 2016, **11**: e144052.

[4] Janelidze S, Mattsson N, Stomrud E, Lindberg O, et al. CSF biomarkers of neuroinflammation and cerebrovascular dysfunction in early Alzheimer disease. Neurology 2018, **91**: e867-e877.

[5] van der Velpen V, Teav T, Gallart-Ayala H, Mehl F, et al. Systemic and central nervous system metabolic alterations in Alzheimer's disease. Alzheimers Res Ther 2019, **11**: 93.

[6] Goudey B, Fung BJ, Schieber C, Faux NG. A blood-based signature of cerebrospinal fluid Aβ(1-42) status. Sci Rep 2019, **9**: 4163.

[7] Chaudhary A, Kushwah S, Maurya NS, Mani A. Insights from RNA-Seq analysis of Alzheimer's data suggest upregulation of GPCRs. Gene Rep 2020, **21**.

[8] Lin PY, Cheng C, Satyanarayanan SK, Chiu LT, et al. Omega-3 fatty acids and blood-based biomarkers in Alzheimer's disease and mild cognitive impairment: A randomized placebo-controlled trial. Brain Behav Immun 2022, **99**: 289-298.

[9] Di Domenico F, Pupo G, Giraldo E, Lloret A, et al. Autoantibodies Profile in Matching CSF and Serum from AD and aMCI patients: Potential Pathogenic Role and Link to Oxidative Damage. Curr Alzheimer Res 2016, **13**: 112-122.

[10] Grammas P, Ovase R. Inflammatory factors are elevated in brain microvessels in Alzheimer's disease. Neurobiol Aging 2001, **22**: 837-842.

[11] Fiala M, Lin J, Ringman J, Kermani-Arab V, et al. Ineffective phagocytosis of amyloid-beta by macrophages of Alzheimer's disease patients. J Alzheimers Dis 2005, **7**: 221-232, 255-262.

[12] Grammas P, Samany PG, Thirumangalakudi L. Thrombin and inflammatory proteins are elevated in Alzheimer's disease microvessels: implications for disease pathogenesis. J Alzheimers Dis 2006, **9**: 51-58.

[13] Weeraratna AT, Kalehua A, Deleon I, Bertak D, et al. Alterations in immunological and neurological gene expression patterns in Alzheimer's disease tissues. Exp Cell Res 2007, **313**: 450-461.

[14] Tripathy D, Thirumangalakudi L, Grammas P. Expression of macrophage inflammatory protein 1-alpha is elevated in Alzheimer's vessels and is regulated by oxidative stress. J Alzheimers Dis 2007, **11**: 447-455.

[15] Man SM, Ma YR, Shang DS, Zhao WD, et al. Peripheral T cells overexpress MIP-1alpha to enhance its transendothelial migration in Alzheimer's disease. Neurobiol Aging 2007, **28**: 485-496.

[16] Wilberding A, Morimoto K, Satoh H, Harano K, et al. Multiple cytokines are involved in the early events leading to the Alzheimer's disease pathology. Tottori Rinsho Kagaku Kenkyukai Shi 2008, **1**: 359-373.

[17] Reale M, Iarlori C, Feliciani C, Gambi D. Peripheral chemokine receptors, their ligands, cytokines and Alzheimer's disease. J Alzheimers Dis 2008, **14**: 147-159.

[18] Liao Y, Guan ZZ, Rivka R. Changes of nuclear factor and inflammatory chemotactic factors in brain of patients with Alzheimer's disease. Chinese Journal of Pathology 2011, **40**: 585-589.

[19] Westman G, Lidehall AK, Magnusson P, Ingelsson M, et al. Decreased proportion of cytomegalovirus specific CD8 T-cells but no signs of general immunosenescence in Alzheimer's disease. Plos One 2013, **8**: e77921.

[20] Goldeck D, Larbi A, Pellicanó M, Alam I, et al. Enhanced Chemokine Receptor Expression on Leukocytes of Patients with Alzheimer's Disease. Plos One 2013, **8**: e66664.

[21] Westman G, Berglund D, Widén J, Ingelsson M, et al. Increased inflammatory response in cytomegalovirus seropositive patients with Alzheimer's disease. Plos One 2014, **9**: e96779.

[22] Julian A, Dugast E, Ragot S, Krolak-Salmon P, et al. There is no correlation between peripheral inflammation and cognitive status at diagnosis in Alzheimer's disease. Aging Clin Exp Res 2015, **27**: 589-594.

[23] Guedes JR, Santana I, Cunha C, Duro D, et al. MicroRNA deregulation and chemotaxis and phagocytosis impairment in Alzheimer's disease. Alzheimers Dement (Amst) 2016, **3**: 7-17.

[24] Le Page A, Lamoureux J, Bourgade K, Frost EH, et al. Polymorphonuclear Neutrophil Functions are Differentially Altered in Amnestic Mild Cognitive Impairment and Mild Alzheimer's Disease Patients. J Alzheimers Dis 2017, **60**: 23-42.

[25] Vérité J, Page G, Paccalin M, Julian A, et al. Differential chemokine expression under the control of peripheral blood mononuclear cells issued from Alzheimer's patients in a human blood brain barrier model. Plos One 2018, **13**: e201232.

[26] Rakic S, Hung Y, Smith M, So D, et al. Systemic infection modifies the neuroinflammatory response in late stage Alzheimer's disease. Acta Neuropathol Commun 2018, **6**: 88.

[27] Sanfilippo C, Castrogiovanni P, Imbesi R, Nunnari G, et al. Postsynaptic damage and microglial activation in AD patients could be linked CXCR4/CXCL12 expression levels. Brain Res 2020, **1749**: 147127.

[28] Bryant AG, Hu M, Carlyle BC, Arnold SE, et al. Cerebrovascular Senescence Is Associated With Tau Pathology in Alzheimer's Disease. Front Neurol 2020, **11**: 575953.

[29] Wu Q, Kong W, Wang S. Peripheral Blood Biomarkers CXCL12 and TNFRSF13C Associate with Cerebrospinal Fluid Biomarkers and Infiltrating Immune Cells in Alzheimer Disease. J Mol Neurosci 2021, **71**: 1485-1494.

[30] Flores-Aguilar L, Iulita MF, Orciani C, Tanna N, et al. Cognitive and brain cytokine profile of non-demented individuals with cerebral amyloid-beta deposition. J Neuroinflammation 2021, **18**: 147.

[31] Asby D, Boche D, Allan S, Love S, et al. Systemic infection exacerbates cerebrovascular dysfunction in Alzheimer's disease. Brain 2021, **144**: 1869-1883.

[32] Sorrentino S, Ascari R, Maderna E, Catania M, et al. Microglial Heterogeneity and Its Potential Role in Driving Phenotypic Diversity of Alzheimer's Disease. Int J Mol Sci 2021, **22**.

[33] Barroeta-Espar I, Weinstock LD, Perez-Nievas BG, Meltzer AC, et al. Distinct cytokine profiles in human brains resilient to Alzheimer's pathology. Neurobiol Dis 2019, **121**: 327-337.

[34] Bradburn S, McPhee J, Bagley L, Carroll M, et al. Dysregulation of C-X-C motif ligand 10 during aging and association with cognitive performance. Neurobiol Aging 2018, **63**: 54-64.

[35] Ashutosh, Kou W, Cotter R, Borgmann K, et al. CXCL8 protects human neurons from amyloid-β-induced neurotoxicity: relevance to Alzheimer's disease. Biochem Biophys Res Commun 2011, **412**: 565-571.

[36] Tripathy D, Thirumangalakudi L, Grammas P. RANTES upregulation in the Alzheimer's disease brain: a possible neuroprotective role. Neurobiol Aging 2010, **31**: 8-16.

[37] Sokolova A, Hill MD, Rahimi F, Warden LA, et al. Monocyte chemoattractant protein-1 plays a dominant role in the chronic inflammation observed in Alzheimer's disease. Brain Pathol 2009, **19**: 392-398.

[38] Taipa R, Das Neves SP, Sousa AL, Fernandes J, et al. Proinflammatory and anti-inflammatory cytokines in the CSF of patients with Alzheimer's disease and their correlation with cognitive decline. Neurobiol Aging 2019, **76**: 125-132.

[39] Elkind M, Moon M, Rundek T, Wright CB, et al. Immune markers are associated with cognitive performance in a multiethnic cohort: The Northern Manhattan Study. Brain Behav Immun 2021, **97**: 186-192.

[40] Gaetani L, Bellomo G, Parnetti L, Blennow K, et al. Neuroinflammation and Alzheimer's Disease: A Machine Learning Approach to CSF Proteomics. Cells 2021, **10**.

[41] Ponomareva EV, Krinsky SA, Gavrilova SI. [Prognosis of amnestic mild cognitive impairment: clinical and immunological correlations]. Zh Nevrol Psikhiatr Im S S Korsakova 2021, **121**: 16-22.

[42] Ferguson SA, Varma V, Sloper D, Panos JJ, et al. Increased inflammation in BA21 brain tissue from African Americans with Alzheimer's disease. Metab Brain Dis 2020, **35**: 121-133.

[43] Thomas AJ, Hamilton CA, Donaghy PC, Martin-Ruiz C, et al. Prospective longitudinal evaluation of cytokines in mild cognitive impairment due to AD and Lewy body disease. Int J Geriatr Psychiatry 2020, **35**: 1250-1259.

[44] Blaabjerg M, Hemdrup AL, Drici L, Ruprecht K, et al. Omics-Based Approach Reveals Complement-Mediated Inflammation in Chronic Lymphocytic Inflammation With Pontine Perivascular Enhancement Responsive to Steroids (CLIPPERS). Front Immunol 2018, **9**: 741.

[45] Bettcher BM, Johnson SC, Fitch R, Casaletto KB, et al. Cerebrospinal Fluid and Plasma Levels of Inflammation Differentially Relate to CNS Markers of Alzheimer's Disease Pathology and Neuronal Damage. J Alzheimers Dis 2018, **62**: 385-397.

[46] Butcher L, Pérès K, André P, Morris RH, et al. Association between plasma CCL11 (eotaxin-1) and cognitive status in older adults: Differences between rural and urban dwellers. Exp Gerontol 2018, **113**: 173-179.

[47] Julian A, Rioux-Bilan A, Ragot S, Krolak-Salmon P, et al. Blood Inflammatory Mediators and Cognitive Decline in Alzheimer's Disease: A Two Years Longitudinal Study. J Alzheimers Dis 2018, **63**: 87-92.

[48] Laugisch O, Johnen A, Maldonado A, Ehmke B, et al. Periodontal Pathogens and Associated Intrathecal Antibodies in Early Stages of Alzheimer's Disease. J Alzheimers Dis 2018, **66**: 105-114.

[49] Trombetta BA, Carlyle BC, Koenig AM, Shaw LM, et al. The technical reliability and biotemporal stability of cerebrospinal fluid biomarkers for profiling multiple pathophysiologies in Alzheimer's disease. Plos One 2018, **13**: e193707.

[50] Abe Y, Kimura N, Takahashi R, Gotou M, et al. Relationship between cytokine levels in the cerebrospinal fluid and 11C-Pittsburgh compound B retention in patients with mild cognitive impairment. Geriatr Gerontol Int 2017, **17**: 1907-1913.

[51] Kimura A, Yoshikura N, Hayashi Y, Inuzuka T. Cerebrospinal Fluid C-C Motif Chemokine Ligand 2 Correlates with Brain Atrophy and Cognitive Impairment in Alzheimer's Disease. Journal of Alzheimer's Disease 2017, **61**: 581-588.

[52] Popp J, Oikonomidi A, Tautvydaitė D, Dayon L, et al. Markers of neuroinflammation associated with Alzheimer's disease pathology in older adults. Brain Behav Immun 2017, **62**: 203-211.

[53] Malashenkova IK, Hailov NA, Krynskiy SA, Ogurtsov DP, et al. [Levels of proinflammatory cytokines and vascular endothelial growth factor in patients with Alzheimer's disease and mild cognitive impairment]. Zh Nevrol Psikhiatr Im S S Korsakova 2016, **116**: 39-43.

[54] Khan W, Aguilar C, Kiddle SJ, Doyle O, et al. A Subset of Cerebrospinal Fluid Proteins from a Multi-Analyte Panel Associated with Brain Atrophy, Disease Classification and Prediction in Alzheimer's Disease. Plos One 2015, **10**: e134368.

[55] Schmitz M, Hermann P, Oikonomou P, Stoeck K, et al. Cytokine profiles and the role of cellular prion protein in patients with vascular dementia and vascular encephalopathy. Neurobiol Aging 2015, **36**: 2597-2606.

[56] Kauwe JS, Bailey MH, Ridge PG, Perry R, et al. Genome-wide association study of CSF levels of 59 alzheimer's disease candidate proteins: significant associations with proteins involved in amyloid processing and inflammation. Plos Genet 2014, **10**: e1004758.

[57] Pyykkö OT, Lumela M, Rummukainen J, Nerg O, et al. Cerebrospinal fluid biomarker and brain biopsy findings in idiopathic normal pressure hydrocephalus. Plos One 2014, **9**: e91974.

[58] Stoeck K, Schmitz M, Ebert E, Schmidt C, et al. Immune responses in rapidly progressive dementia: A comparative study of neuroinflammatory markers in Creutzfeldt-Jakob disease, Alzheimer's disease and multiple sclerosis. J Neuroinflammation 2014, **11**.

[59] Stoeck K, Schmitz M, Schmidt C, Zerr I. Immune responses in rapid dementia: A comparative study on neuroinflammatory markers in CJD, AD, rpAD and MS patients. Prion 2013, **7**: 58.

[60] Craig-Schapiro R, Kuhn M, Xiong C, Pickering EH, et al. Multiplexed immunoassay panel identifies novel CSF biomarkers for Alzheimer's disease diagnosis and prognosis. Plos One 2011, **6**: e18850.

[61] Hu WT, Chen-Plotkin A, Grossman M, Arnold SE, et al. Novel CSF biomarkers for frontotemporal lobar degenerations. Neurology 2010, **75**: 2079-2086.

[62] Kang SL, Ji HC, Kyung HL, Shin MJ, et al. Plasma levels of monocyte chemotactic protein 3 and beta-nerve growth factor increase with amnestic mild cognitive impairment. Cellular and Molecular Immunology 2009, **6**: 143-147.

[63] Jefferson AL, Massaro JM, Wolf PA, Seshadri S, et al. Inflammatory biomarkers are associated with total brain volume: the Framingham Heart Study. Neurology 2007, **68**: 1032-1038.

[64] Zuliani G, Guerra G, Ranzini M, Rossi L, et al. High interleukin-6 plasma levels are associated with functional impairment in older patients with vascular dementia. Int J Geriatr Psychiatry 2007, **22**: 305-311.

[65] Sun YX, Minthon L, Wallmark A, Warkentin S, et al. Inflammatory markers in matched plasma and cerebrospinal fluid from patients with Alzheimer's disease. Dement Geriatr Cogn Disord 2003, **16**: 136-144.

[66] Geppert AM, Losy J, Przedpelska-Ober E, Kozubski W. CCL3 correlates with the number of mood disturbances and personality changes in patients with Alzheimer's disease. Psychiatry Res 2010, **176**: 261-264.

[67] Mulugeta E, Molina-Holgado F, Elliott MS, Hortobagyi T, et al. Inflammatory mediators in the frontal lobe of patients with mixed and vascular dementia. Dement Geriatr Cogn Disord 2008, **25**: 278-286.

[68] Baune BT, Ponath G, Golledge J, Varga G, et al. Association between IL-8 cytokine and cognitive performance in an elderly general population--the MEMO-Study. Neurobiol Aging 2008, **29**: 937-944.

[69] Harries LW, Bradley-Smith RM, Llewellyn DJ, Pilling LC, et al. Leukocyte CCR2 expression is associated with mini-mental state examination score in older adults. Rejuvenation Res 2012, **15**: 395-404.

[70] Goldstein FC, Zhao L, Steenland K, Levey AI. Inflammation and cognitive functioning in African Americans and Caucasians. Int J Geriatr Psychiatry 2015, **30**: 934-941.

[71] de Pablo-Bernal RS, Cañizares J, Rosado I, Galvá MI, et al. Monocyte Phenotype and Polyfunctionality Are Associated With Elevated Soluble Inflammatory Markers, Cytomegalovirus Infection, and Functional and Cognitive Decline in Elderly Adults. J Gerontol a Biol Sci Med Sci 2016, **71**: 610-618.

[72] Bettcher BM, Fitch R, Wynn MJ, Lalli MA, et al. MCP-1 and eotaxin-1 selectively and negatively associate with memory in MCI and Alzheimer's disease dementia phenotypes. Alzheimer's & dementia : diagnosis, assessment & disease monitoring 2016, **3**: 91-97.

[73] Stenfors C, Jonsdottir IH, Magnusson HL, Theorell T. Associations between systemic pro-inflammatory markers, cognitive function and cognitive complaints in a population-based sample of working adults. J Psychosom Res 2017, **96**: 49-59.

[74] Kimura A, Takemura M, Saito K, Yoshikura N, et al. Comparison of cerebrospinal fluid profiles in Alzheimer's disease with multiple cerebral microbleeds and cerebral amyloid angiopathy-related inflammation. J Neurol 2017, **264**: 373-381.

[75] Johansson P, Almqvist EG, Wallin A, Johansson JO, et al. Reduced cerebrospinal fluid concentration of interleukin-12/23 subunit p40 in patients with cognitive impairment. Plos One 2017, **12**: e176760.

[76] Cattaneo A, Cattane N, Galluzzi S, Provasi S, et al. Association of brain amyloidosis with pro-inflammatory gut bacterial taxa and peripheral inflammation markers in cognitively impaired elderly. Neurobiol Aging 2017, **49**: 60-68.

[77] Roos P, von Essen MR, Nielsen TT, Johannsen P, et al. Inflammatory markers of CHMP2B-mediated frontotemporal dementia. J Neuroimmunol 2018, **324**: 136-142.

[78] Kim JW, Stewart R, Kang HJ, Bae KY, et al. Longitudinal Associations Between Serum Cytokine Levels and Dementia. Front Psychiatry 2018, **9**: 606.

[79] Tsai CL, Sun HS, Kuo YM, Pai MC. The Role of Physical Fitness in Cognitive-Related Biomarkers in Persons at Genetic Risk of Familial Alzheimer's Disease. J Clin Med 2019, **8**.

[80] Bettcher BM, Neuhaus J, Wynn MJ, Elahi FM, et al. Increases in a Pro-inflammatory Chemokine, MCP-1, Are Related to Decreases in Memory Over Time. Front Aging Neurosci 2019, **11**.

[81] Serre-Miranda C, Roque S, Santos NC, Costa P, et al. Cognition Is Associated With Peripheral Immune Molecules in Healthy Older Adults: A Cross-Sectional Study. Front Immunol 2020, **11**: 2045.

[82] Murcia J, Weinert A, Freitas C, Arens DK, et al. Atypical chemokine receptor ACKR2-V41A has decreased CCL2 binding, scavenging, and activation, supporting sustained inflammation and increased Alzheimer's disease risk. Sci Rep 2020, **10**: 8019.

[83] Motta C, Finardi A, Toniolo S, Di Lorenzo F, et al. Protective Role of Cerebrospinal Fluid Inflammatory Cytokines in Patients with Amnestic Mild Cognitive Impairment and Early Alzheimer's Disease Carrying Apolipoprotein E4 Genotype. J Alzheimers Dis 2020, **76**: 681-689.

[84] Bawa KK, Krance SH, Herrmann N, Cogo-Moreira H, et al. A peripheral neutrophil-related inflammatory factor predicts a decline in executive function in mild Alzheimer's disease. J Neuroinflammation 2020, **17**: 84.

[85] Tsai CL, Erickson KI, Sun HS, Kuo YM, et al. A cross-sectional examination of a family history of Alzheimer's disease and ApoE epsilon 4 on physical fitness, molecular biomarkers, and neurocognitive performance. Physiol Behav 2021, **230**: 113268.

[86] Albrecht DS, Sagare A, Pachicano M, Sweeney MD, et al. Early neuroinflammation is associated with lower amyloid and tau levels in cognitively normal older adults. Brain Behav Immun 2021, **94**: 299-307.

[87] Aksnes M, Aass H, Tiiman A, Edwin TH, et al. Associations of cerebrospinal fluid amyloidogenic nanoplaques with cytokines in Alzheimer's disease. Transl Neurodegener 2021, **10**: 18.

[88] Galimberti D, Schoonenboom N, Scarpini E, Scheltens P. Chemokines in serum and cerebrospinal fluid of Alzheimer's disease patients. Ann Neurol 2003, **53**: 547-548.

[89] Huerta C, Álvarez V, Mata IF, Coto E, et al. Chemokines (RANTES and MCP-1) and chemokine-receptors (CCR2 and CCR5) gene polymorphisms in Alzheimer's and Parkinson's disease. Neurosci Lett 2004, **370**: 151-154.

[90] Tamura Y, Sakasegawa Y, Omi K, Kishida H, et al. Association study of the chemokine, CXC motif, ligand 1 (CXCL1) gene with sporadic Alzheimer's disease in a Japanese population. Neurosci Lett 2005, **379**: 149-151.

[91] Venturelli E, Galimberti D, Fenoglio C, Lovati C, et al. Candidate gene analysis of IP-10 gene in patients with Alzheimer's disease. Neurosci Lett 2006, **404**: 217-221.

[92] BALISTRERI CR, GRIMALDI MP, VASTO S, LISTI F, et al. Association between the Polymorphism of CCR5 and Alzheimer's Disease: Results of a Study Performed on Male and Female Patients from Northern Italy. Ann N Y Acad Sci 2006, **1089**: 454-461.

[93] Galimberti D, Venturelli E, Fenoglio C, Lovati C, et al. IP-10 serum levels are not increased in mild cognitive impairment and Alzheimer's disease. Eur J Neurol 2007, **14**: e3-e4.

[94] Villa C, Venturelli E, Fenoglio C, Clerici F, et al. CCL8/MCP-2 association analysis in patients with Alzheimer's disease and frontotemporal lobar degeneration. J Neurol 2009, **256**: 1379-1381.

[95] Kester MI, van der Flier WM, Visser A, Blankenstein MA, et al. Decreased mRNA expression of CCL5 [RANTES] in Alzheimer's disease blood samples. Clin Chem Lab Med 2011, **50**: 61-65.

[96] Furney SJ, Kronenberg D, Simmons A, Güntert A, et al. Combinatorial markers of mild cognitive impairment conversion to Alzheimer's disease-cytokines and MRI measures together predict disease progression. 2011, 2: 789-799.

[97] Soares HD, Potter WZ, Pickering E, Kuhn M, et al. Plasma biomarkers associated with the apolipoprotein E genotype and Alzheimer disease. Arch Neurol 2012, **69**: 1310-1317.

[98] Doecke JD, Laws SM, Faux NG, Wilson W, et al. Blood-based protein biomarkers for diagnosis of Alzheimer disease. Arch Neurol 2012, **69**: 1318-1325.

[99] Saresella M, Marventano I, Calabrese E, Piancone F, et al. A complex proinflammatory role for peripheral monocytes in Alzheimer's disease. J Alzheimers Dis 2014, **38**: 403-413.

[100] Schmidt C, Wolff M, von Ahsen N, Lange K, et al. CR1 is potentially associated with rate of decline in sporadic Alzheimer's disease. J Clin Neurosci 2014, **21**: 1705-1708.

[101] Flex A, Giovannini S, Biscetti F, Liperoti R, et al. Effect of proinflammatory gene polymorphisms on the risk of Alzheimer's disease. Neurodegener Dis 2014, **13**: 230-236.

[102] Strobel S, Grünblatt E, Riederer P, Heinsen H, et al. Changes in the expression of genes related to neuroinflammation over the course of sporadic Alzheimer's disease progression: CX3CL1, TREM2, and PPARγ. J Neural Transm (Vienna) 2015, **122**: 1069-1076.

[103] Lalli MA, Bettcher BM, Arcila ML, Garcia G, et al. Whole-genome sequencing suggests a chemokine gene cluster that modifies age at onset in familial Alzheimer's disease. Mol Psychiatry 2015, **20**: 1294-1300.

[104] Kang HJ, Kim JM, Kim SW, Shin IS, et al. Associations of cytokine genes with Alzheimer's disease and depression in an elderly Korean population. J Neurol Neurosurg Psychiatry 2015, **86**: 1002-1007.

[105] Melah KE, Lu SY, Hoscheidt SM, Alexander AL, et al. Cerebrospinal Fluid Markers of Alzheimer's Disease Pathology and Microglial Activation are Associated with Altered White Matter Microstructure in Asymptomatic Adults at Risk for Alzheimer's Disease. J Alzheimers Dis 2016, **50**: 873-886.

[106] Vakilian A, Razavi-Nasab SM, Ravari A, Mirzaei T, et al. Vitamin B12 in Association with Antipsychotic Drugs Can Modulate the Expression of Pro-/Anti-Inflammatory Cytokines in Alzheimer Disease Patients. Neuroimmunomodulation 2017, **24**: 310-319.

[107] Ott BR, Jones RN, Daiello LA, de la Monte SM, et al. Blood-cerebrospinal fluid barrier gradients in mild cognitive impairment and Alzheimer's disease: Relationship to inflammatory cytokines and chemokines. Front Aging Neurosci 2018, **10**.

[108] López-López A, Gelpi E, Lopategui DM, Vidal-Taboada JM. Association of the CX3CR1-V249I Variant with Neurofibrillary Pathology Progression in Late-Onset Alzheimer's Disease. Mol Neurobiol 2018, **55**: 2340-2349.

[109] Bowman GL, Dayon L, Kirkland R, Wojcik J, et al. Blood‐brain barrier breakdown, neuroinflammation, and cognitive decline in older adults. Alzheimer's & Dementia 2018, **14**: 1640-1650.

[110] Iulita MF, Ganesh A, Pentz R, Flores AL, et al. Identification and Preliminary Validation of a Plasma Profile Associated with Cognitive Decline in Dementia and At-Risk Individuals: A Retrospective Cohort Analysis. J Alzheimers Dis 2019, **67**: 327-341.

[111] Wee JJ, Kumar S. Prediction of hub genes of Alzheimer's disease using a protein interaction network and functional enrichment analysis. Genomics Inform 2020, **18**: e39.

[112] Pillai JA, Bena J, Bebek G, Bekris LM, et al. Inflammatory pathway analytes predicting rapid cognitive decline in MCI stage of Alzheimer's disease. Ann Clin Transl Neurol 2020, **7**: 1225-1239.

[113] Peters VTA, Verbeek MM, Alkema W, Pickkers P, et al. Downregulation of synapse-associated protein expression and loss of homeostatic microglial control in cerebrospinal fluid of infectious patients with delirium and patients with Alzheimer's disease. Brain Behav Immun 2020, **89**: 656-667.

[114] Elahi FM, Casaletto KB, La Joie R, Walters SM, et al. Plasma biomarkers of astrocytic and neuronal dysfunction in early- and late-onset Alzheimer's disease. Alzheimers Dement 2020, **16**: 681-695.

[115] Cicero CE, Donzuso G, Luca A, Davi M, et al. Midbrain MRI morphometric measurements and MCI in Parkinson's disease: the PACOS study. Eur J Neurol 2020, **27**: 184.

[116] El IF, Gressier B, Devos D, Belarbi K. A Computational Exploration of the Molecular Network Associated to Neuroinflammation in Alzheimer's Disease. Front Pharmacol 2021, **12**: 630003.

[117] Delaby C, Julian A, Page G, Ragot S, et al. NFL strongly correlates with TNF-R1 in the plasma of AD patients, but not with cognitive decline. Sci Rep 2021, **11**: 10283.

[118] Chai YL, Chong JR, Raquib AR, Xu X, et al. Plasma osteopontin as a biomarker of Alzheimer's disease and vascular cognitive impairment. Sci Rep 2021, **11**: 4010.

[119] Xie K, Qin Q, Long Z, Yang Y, et al. High-Throughput Metabolomics for Discovering Potential Biomarkers and Identifying Metabolic Mechanisms in Aging and Alzheimer's Disease. Front Cell Dev Biol 2021, **9**: 602887.

[120] Olson L, Humpel C. Growth factors and cytokines/chemokines as surrogate biomarkers in cerebrospinal fluid and blood for diagnosing Alzheimer's disease and mild cognitive impairment. Exp Gerontol 2010, **45**: 41-46.

[121] Liu C, Guo HD, Kang XP. [Effects of chemokines on the occurrence and development of Alzheimer's disease]. Sheng Li Ke Xue Jin Zhan 2014, **45**: 125-128.

[122] Laske C, Stellos K, Stransky E, Seizer P, et al. Decreased plasma and cerebrospinal fluid levels of stem cell factor in patients with early Alzheimer's disease. J Alzheimers Dis 2008, **15**: 451-460.

[123] Rocha DPM, Gómez RM, Berretta R, Moscato P. Differences in abundances of cell-signalling proteins in blood reveal novel biomarkers for early detection of clinical Alzheimer's disease. Plos One 2011, **6**: e17481.

[124] King E, O'Brien J, Donaghy P, Williams-Gray CH, et al. Inflammation in mild cognitive impairment due to Parkinson's disease, Lewy body disease, and Alzheimer's disease. Int J Geriatr Psychiatry 2019, **34**: 1244-1250.

[125] Schipke CG, Menne F, Rubow S, Sigle JP, et al. Value of a Panel of 6 Serum Biomarkers to Differentiate Between Healthy Controls and Mild Cognitive Impairment Due to Alzheimer Disease. Alzheimer Dis Assoc Disord 2020, **34**: 318-324.

[126] Rodríguez-Borja E, Monge-Argilés JA, Soriano-Terol MA, Muñoz-Ruiz C. Multiplex analysis of intrathecal cytokines in patients with mild cognitive impairment. Revista del Laboratorio Clinico 2011, **4**: 177-185.

[127] Hu WT, Chen-Plotkin A, Arnold SE, Grossman M, et al. Novel CSF biomarkers for Alzheimer's disease and mild cognitive impairment. Acta Neuropathol 2010, **119**: 669-678.

[128] Cherry JD, Stein TD, Tripodis Y, Alvarez VE, et al. CCL11 is increased in the CNS in chronic traumatic encephalopathy but not in Alzheimer's disease. Plos One 2017, **12**: e185541.

[129] Kim SM, Song J, Kim S, Han C, et al. Identification of peripheral inflammatory markers between normal control and Alzheimer's disease. Bmc Neurol 2011, **11**: 51.
